# Analyses of the folding sites of irregular β-trefoil fold proteins through sequence-based techniques and Gō-model simulations

**DOI:** 10.1186/s12860-020-00271-4

**Published:** 2020-07-21

**Authors:** Risako Kimura, Panyavut Aumpuchin, Shoya Hamaue, Takumi Shimomura, Takeshi Kikuchi

**Affiliations:** 1 Department of Bioinformatics, College of Life Sciences, Ritsumeikan University, 1-1-1 Nojihigashi, Kusatsu, Shiga 525-8577 Japan; 2 National Center for Genetic Engineering and Biotechnology (BIOTEC), 113 Thailand Science Park, Phaholyothin Road, Klong Luang, Pathumthani 12120 Thailand

**Keywords:** β-Trefoil fold, Folding mechanism, Inter-residue average distance statistics, Conserved hydrophobic residues, Gō-model simulation

## Abstract

**Background:**

The details of the folding mechanisms have not yet been fully understood for many proteins, and it is believed that the information on the folding mechanism of a protein is encoded in its amino acid sequence. β-trefoil proteins are known to have the same 3D scaffold, namely, a three-fold symmetric scaffold, despite the proteins’ low sequence identity among superfamilies. In this study, we extract an initial folding unit from the amino acid sequences of irregular β-trefoil proteins by constructing an average distance map (ADM) and utilizing inter-residue average distance statistics to determine the relative contact frequencies for residue pairs in terms of F values. We compare our sequence-based prediction results with the packing between hydrophobic residues in native 3D structures and a Gō-model simulation.

**Results:**

The ADM and F-value analyses predict that the N-terminal and C-terminal regions are compact and that the hydrophobic residues at the central region can be regarded as an interaction center with other residues. These results correspond well to those of the Gō-model simulations. Moreover, our results indicate that the irregular parts in the β-trefoil proteins do not hinder the protein formation. Conserved hydrophobic residues on the β5 strand are always the interaction center of packing between the conserved hydrophobic residues in both regular and irregular β-trefoil proteins.

**Conclusions:**

We revealed that the β5 strand plays an important role in β-trefoil protein structure construction. The sequence-based methods used in this study can extract the protein folding information from only amino acid sequence data, and well corresponded to 3D structure-based Gō-model simulation and available experimental results.

## Background

**Fig. 1:**
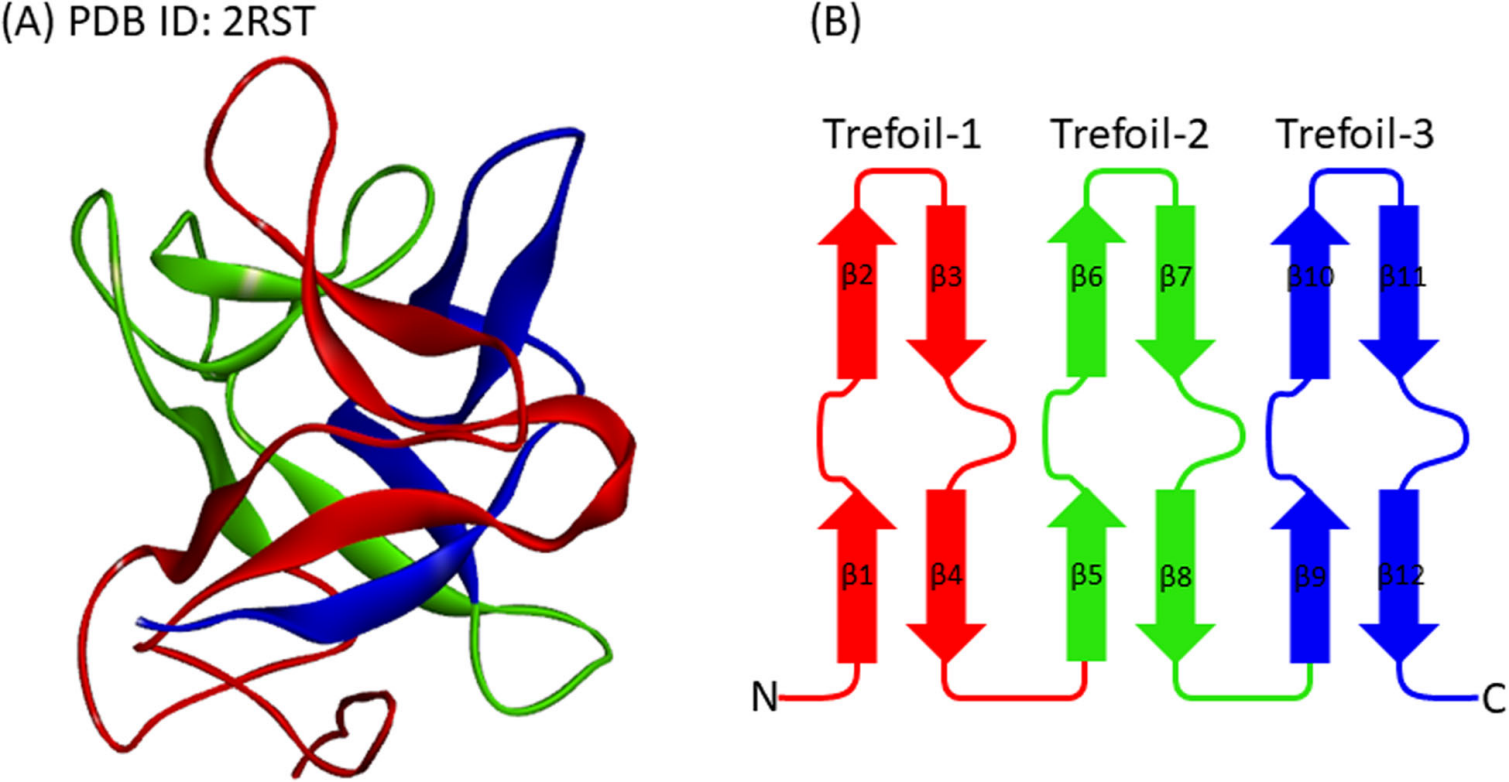
The common 3D structure of high symmetric β-trefoil fold protein. **a** 3D structure of galactose-binding lectin (PDB ID: 2RST). The number of residues is 132. The three β-trefoil units are colored in red, green, and blue, respectively, from the N- to C-termini. **b** Schematic drawing of β-trefoil topology. β-strands colored in orange and green form a barrel and the cap structures, respectively

A β-trefoil protein exhibits pseudo three-fold symmetry and is observed widely in the protein 3D structure space. We show the 3D structure and schematic drawings of the three symmetrical units of cytokine (2K8R) as a representative β-trefoil protein in Fig. 1a and b. How a β-trefoil protein folds into such a highly symmetrical structure is an interesting problem and has been studied by various researchers [1–4]. We also clarified how the folding information of such a symmetrical β-trefoil protein is encoded in its amino acid sequence in the previous study [5]. However, there are some β-trefoil proteins containing irregular structures. In such a protein, the three-fold symmetry is partly disturbed. It is fascinating that whether a β-trefoil protein with an irregular structure folds into its native structure via the same folding pathway as a β-trefoil protein with high three-fold symmetry.

In the present study, we investigate the relationship between the folding mechanisms of β-trefoil proteins with irregular structures and their amino acid sequences. We utilize the same techniques as in our previous study, [5] in which we used inter-residue average distance statistics to generate a predicted contact map and perform contact frequency analysis of residues in a protein in a random state to identify which residues are significant for folding into the characteristic β-trefoil scaffold. So far, we have confirmed that these methods, in combination with information about the conserved hydrophobic residues, can predict the folding properties for the following proteins: fatty-acid-binding proteins, [6] globin-like fold proteins, [7] IgG-binding and albumin-binding domains, [8] Ig-like fold proteins, [9, 10] ferredoxin-like fold proteins, [11] β-trefoil fold proteins, [5] and lysozyme-like superfamily proteins [12].

We also focus on the hydrophobic packing formed by conserved hydrophobic residues in the native structures and compare the packing hydrophobic residues and the residues significant in folding. To confirm the results that we predict from the amino acid sequence information, we conduct Gō-model simulations. From the results obtained, we identify the residues significant for irregular β-trefoil proteins to fold into the 3D structures.

In the present study, we apply the coarse-grained Gō model which we have developed [13–15]. This technique incorporates the effects of side chains implicitly into a coarse-grained Gō model [13]. It is well-known that a Gō model technique can reproduce the various properties of folding mechanisms of proteins rather precisely such as the relationship between topology and folding rate of a protein, [16–18] the presence or absence of folding intermediates [19–21] and the folding pathways [19, 22, 23]. It has been also demonstrated that our Gō model reproduces the experimentally observed folding processes of SH3 domain, GB proteins and ferredoxin [13–15]. A molecular dynamics (MD) simulation is also widely used to analyze the folding of a protein, but it is applied for a protein with several ten residues. On the other hand, a β-trefoil protein contains about 200 residues. Thus, we think that the size of a β-trefoil protein is relatively too large to apply the MD technique to simulate precisely the folding of a β-trefoil protein.

## Methods

### Proteins treated in this study

**Fig. 2:**
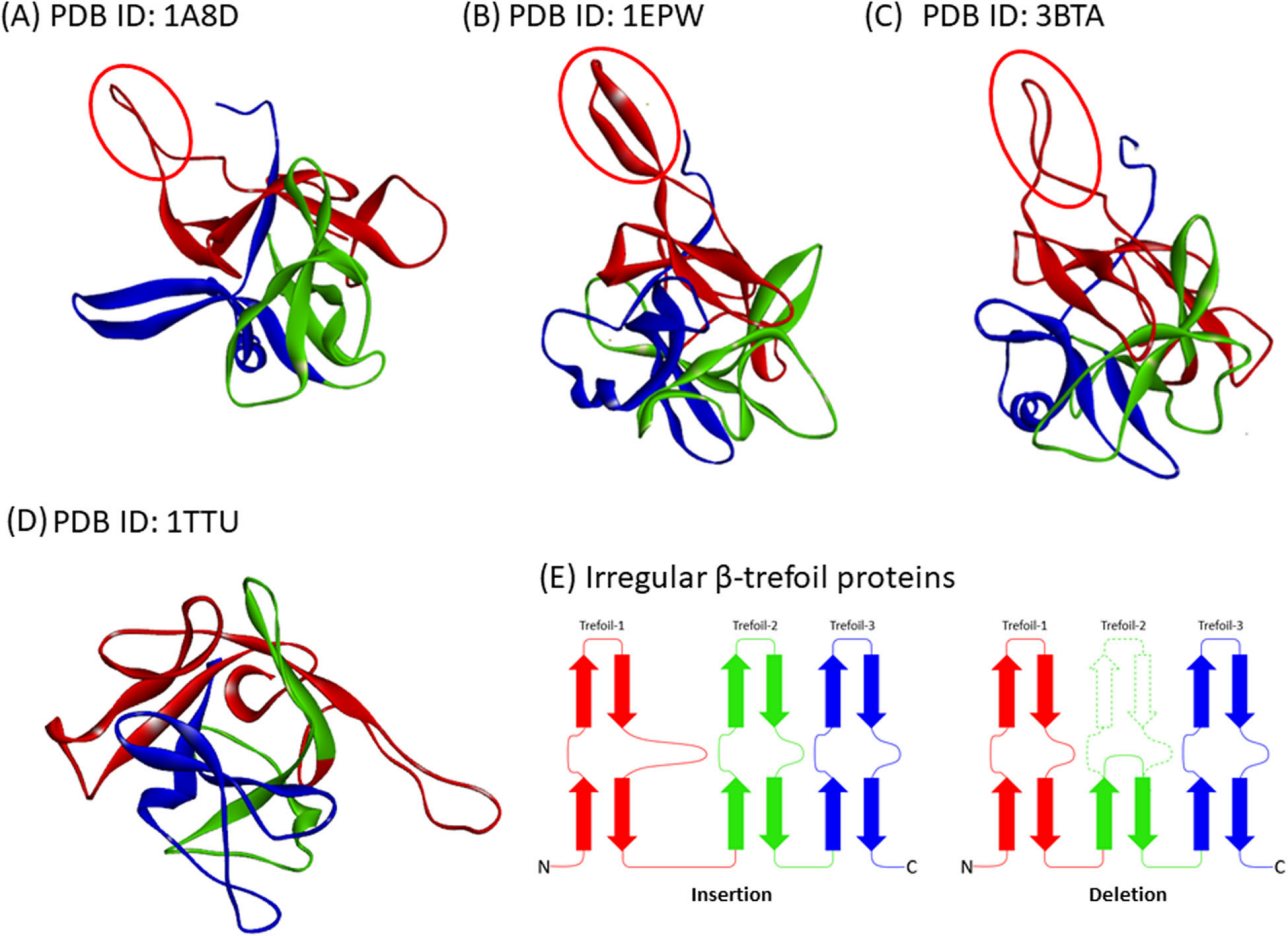
The 3D structure of irregular β-trefoil fold proteins used in this study. **a** 3D structure of Tetanus toxin (PDB ID: 1A8D). **b** 3D structure of Clostridium neurotoxin type B (PDB ID: 1EPW). **c** 3D structure of Botulinum neurotoxin serotype A (PDB ID: 3BTA). **d** 3D structure of CSL bound to DNA (PDB ID: 1TTU). The segment enclosed by a red circle indicates the inserted part. **e** Schematic drawing of two difference irregular structures

In our previous study, we examined regularly structured single-domain proteins of up to 180 residues. That is, we excluded proteins in the STI-like, DNA-binding protein LAG-1 (CSL), and Agglutinin superfamilies. In this study, we selected trefoil proteins containing approximately 180 residues with irregular structures (due to insertion or deletion) from either the STI-like superfamily and the DNA-binding protein LAG-1 (CSL) superfamily: Tetanus toxin (PDB ID (https://www.rcsb.org/) [24]: 1A8D), Clostridium neurotoxin type B (PDB ID: 1EPW), and Botulinum neurotoxin serotype A (PDB ID: 3BTA) in the STI-like superfamily and CSL bound to DNA (PDB ID: 1TTU) in the DNA-binding protein LAG-1 (CSL) superfamily [25–28]. The first three proteins, 1A8D, 1EPW, and 3BTA, were classified to be a pathogenic neurotoxin protein which detected in *Clostridium sp.* While 1TTU was detected in transparent nematode, *Caenorhabditis elegans,* which classified as a part of CSL protein and associated with cell to cell communication. These four 3D structures are illustrated in Fig. 2. The sequence identities are not so high, up to about 30%. The labels of the β-strands in a symmetrical β-trefoil, that is, β1-β12, are kept in the present study. In 1A8D, 1EPW, and 3BTA, a large loop is inserted between β3 and β4. In 1TTU, strands corresponding to β6 and β7 in the central β-trefoil unit are missing. The insertion or deletion causes the partial destruction of three-fold trefoil symmetry. Furthermore, additional 26 high symmetry β-trefoil proteins from our previous study [5] are also derived for the evolutionary analysis study. The information of all 30 target proteins is provided in Table 1.

**Table 1:** Thirty target proteins selected for this study

Superfamily	PDB ID	UniProt ID	Protein name (UniProtKB)	Sequence length
Cytokine	2K8R	P05230	Fibroblast growth factor 1	133
	1Q1U	P61328	Fibroblast growth factor 12	138
	2FDB_M	P55075	Fibroblast growth factor 8	147
	1QQK	Q02195	Fibroblast growth factor 7	129
	1J0S	Q14116	Interleukin-18	157
	6I1B	P01584	Interleukin-1 beta (*Homo sapiens* [*Human*])	153
	1MD6	Q9QYY1	Interleukin-36 receptor antagonist protein	154
	2KKI	P01583	Interleukin-1 alpha	151
	2WRY	O73909	Interleukin-1 beta (*Gallus gallus* [*Chicken*])	155
	2P39	Q9GZV9	Fibroblast growth factor 23	142
	2P23	O95750	Fibroblast growth factor 19	136
Ricin B-like lectins	2RST	O96048	29-kDa galactose-binding lectin	132
	1SR4_A	O06522	Cytolethal distending toxin subunit A	167
	1SR4_C	O06524	Cytolethal distending toxin subunit C	154
	1KNM	P26514	Endo-1,4-beta-xylanase A	129
	1DQG	Q61830	Macrophage mannose receptor 1	134
STI-like	3BX1_D	P07596	Alpha-amylase/subtilisin inhibitor	181
	1TIE	P09943	Trypsin inhibitor DE-3	166
	1R8N	P83667	Kunitz-type serine protease inhibitor DrTI	185
	1WBA	P15465	Albumin-1	171
	2GZB	P83051	Kunitz-type proteinase inhibitor BbCI	164
	3ZC8	D2YW43	Trypsin inhibitor	182
	3TC2	Q8S380	KTI-B protein	181
	1A8D^a^	P04958	Tetanus toxin	205
	1EPW^a^	P10844	Clostridium neurotoxin type B	211
	3BTA^a^	P0DPI1	Botulinum neurotoxin serotype A	204
Actin-crosslinking proteins	1HCD	P13231	Hisactophilin-1	118
MIR domain	1T9F	O61793	Protein R12E2.13 (*Caenorhabditis elegans*)	176
	3HSM	P11716	Ryanodine receptor 1	164
DNA-binding protein LAG-1 (CSL)	1TTU^a^	Q8MXE7	CSL bound to DNA	161

^a^The irregular beta-trefoil proteins

### Average distance map analysis method

An average distance map (ADM) is a kind of predicted contact map constructed from the inter-residue average distance statistics, which is created using only the amino acid sequence information of a protein. This method was originally developed to predict the location of structural domains in a protein [29]. The method is described in detail in a previous study [29]. The following text provides a summary of the method.

### The calculations of inter-residue average distances

The inter-residue average distance for a pair of residues refers to the inter-Cα atomic distance between these residues. Every pair of amino acid type were considered in the calculations of the inter-residue average distances. The separation of the two residues along the amino acid sequence of a protein was considered in calculating the average distance of a residue pair. This separation is called the “range”. The ranges are defined as follows: M = 1 when 1 ≤ k ≤ 8, M = 2 when 9 ≤ k ≤ 20, M = 3 when 21 ≤ k ≤ 30, M = 4 when 31 ≤ k ≤ 40, and so on, where k = |i - j| and M is a range. That is, in each range, the average distances for all pairs of all amino acid types were calculated. Let d(A,B,M) be the average distance of amino acid types A and B in the range M.

### The cutoff distance for each range M

When d(A,B,M) is less than the cutoff distance previously determined for the range M, a plot is made on the map. The set of cutoff distances for the ranges is defined by the following equation:

1 $$ P{(M)}_C=\left(\frac{D}{M}\right)P{(M)}_t, $$

where P(M)_t_, P(M)_c_, and D are the total number of residue pairs with statistically significant average distances, [29] P(M)_c_ is the number of residue pairs with statistically significant average distances smaller than the cutoff distance for M, and D is an adjustable parameter that provides the average plot density of the corresponding ADM close to that of the contact map constructed based on the spatial distances calculated from the actual 3D structure of a protein (real distance map, RDM), respectively. The average plot density is approximated by the following formula: [29].

2 $$ {\rho}_{av}=\frac{C}{N}. $$

Here, N is the total number of residues in a protein and C is the constant value derived from our previous study [29]. The plot density of the RDM of a protein with N residues, constructed with a cutoff distance of 15 Å, is close to *ρ*_*av*_ when we use C = 36.12 [29]. To construct an ADM for a protein, a plot is made on a map when the average distance of a residue pair in a protein is smaller than P(M)_c_. Thus, a map is derived from only the amino acid sequence of a protein based on the inter-residue average distance statistics.

### Prediction of a compact region

**Fig. 3:**
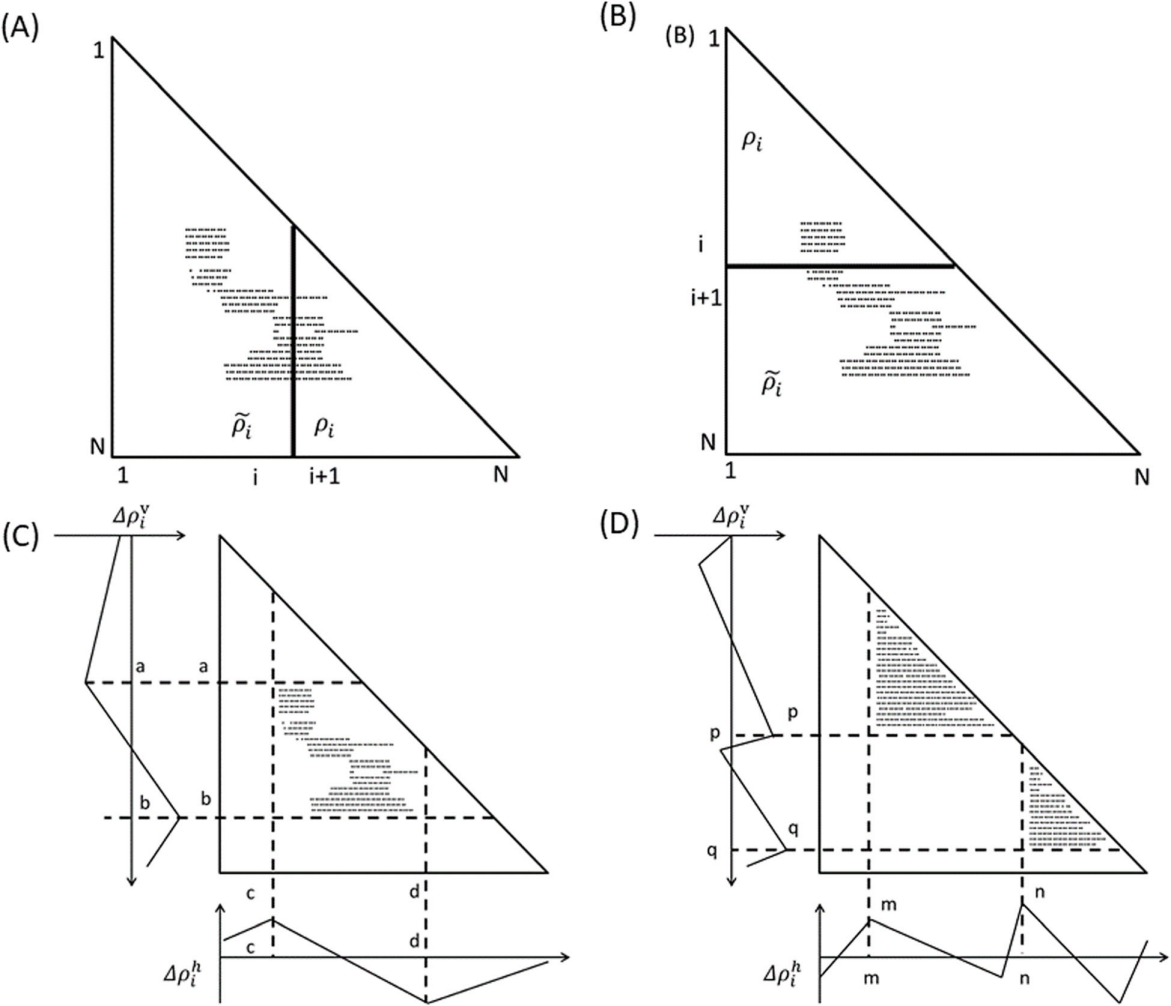
Schematic drawing of an ADM. A line parallel to the y-axis at the i-th residue divides the map into two parts (**a**), and a line parallel to the x-axis at the i-th residue divides the map into two parts (**b**). The density of the plots in the trapezoidal part and the triangular parts are denoted by *ρ*_*i*_ and ${\overset{\sim }{\rho}}_i.$ A hypothetical map with two compact areas near the diagonal is shown, along with the horizontal and vertical scanning plots. This map predicts the existence of two domains at the regions p–q and m–n. We define η as a measure of the compactness of the region, namely, η = $\Delta  {\rho}_m^h+\Delta  {\rho}_p^v$ and η = $\Delta  {\rho}_n^h+\Delta  {\rho}_q^v$

With the constructed ADM for a given protein, a compact region in the protein is predicted as follows. First, the plot density differences in the map are calculated. A density difference means the difference in plot density values between the triangular and trapezoidal parts of the ADM, $\Delta  {\rho}_i={\rho}_i-{\overset{\sim }{\rho}}_i.$ These two parts are defined by a line parallel to the y-axis at the i-th residue or by a line parallel to the x-axis at the i-th residue, as shown in Fig. 3a and b. *∆ρ*_*i*_ are estimated from residue 1 to the total number of residues in a given protein. The plots obtained by the line parallel to the x-axis are called vertical scanning, and those obtained by the line parallel to the y-axis is called horizontal scanning. The v of $\Delta  {\rho}_i^v$ and the h of $\Delta  {\rho}_i^h$ denote the vertical and the horizontal divisions of a map, respectively. In Fig. 3c, the schematic drawing of the vertical and horizontal scanning plots of an ADM is presented.

The detection of a compact region’s boundaries is made based on $\Delta  {\rho}_i^h$ and $\Delta  {\rho}_j^h$. In Fig. 3c, a schematic example of the horizontal scanning plot of $\Delta  {\rho}_i^h$ from 1 to N is presented. In this figure, a peak and a valley appear at c and d, respectively, indicating a large change in plot density values. In the same way, a peak and a valley appear at a and b, respectively (shown on the left of the figure), in the vertical scanning plot of $\Delta  {\rho}_i^v$. This figure indicates that the boundaries of a compact region are evident as a highly dense region of plots, which can be detected by a peak and a valley appearing in the horizontal and vertical scanning plots of the density differences. Finally, a compact region can be predicted by the boundaries defined above, that is, the position of peaks can pinpoint a possible compact region on an ADM as schematically indicated in Fig. 3d. This figure shows a hypothetical ADM with two compact regions near the diagonal. The horizontal and vertical scanning plots show the peaks at residues m and n and residues p and q, and these regions m-p and n-q on the map can be predicted as possible compact regions in a given protein. Furthermore, we use $\eta =\varDelta {\rho}_m^h+\varDelta {\rho}_n^v$ as a measure of the compactness of m-p [29].

A region with a high density of plots, with a high ƞ value, is regarded as a predicted compact region in a 3D structure. We also regard the predicted region as compact in the early stage of folding.

### Secondary structure-based multiple sequence alignment

Multiple sequence alignment provides valuable information about many protein characteristics, including conserved regions which might be related to 3D structure or functional property.

In general, the sequence identities of β-trefoil proteins from different superfamilies are relatively low, making accurate multiple alignments based on only sequences difficult. To detect the relationships among sequences with relatively low sequence identity, this study utilized a multiple sequence alignment technique using secondary structure information, the Combinatorial Extension [30] program in STRAP software [31].

### Evolutionary analyses

Sequence conservation is provided by evolutionary pressure to maintain a given structure and/or structure functions [32, 33]. In this study, the conservation of hydrophobic residues is considered because of their important role in the formation of a protein’s core structure [32]. Ala, Phe, Ile, Leu, Met, Val, Tyr, and Trp are regarded as hydrophobic residues. The conservation of hydrophobic residues means that the above eight hydrophobic residues comprise more than 90% of the residues at an aligned site. A “site” is referred to as the common sequential number in the multiple alignment.

### Conservation of predicted compact regions

The similarity of the location of the predicted compact regions can be regarded as conservation of the predicted compact regions during molecular evolution. A multiple sequence alignment is used to define the conservation of the regions predicted by the ADMs. The procedure is as follows.

The number of residues at a given site that are commonly included in the predicted compact regions is counted to calculate the conserved ratio (this number to the number of aligned sequences). We then make a histogram of this ratio versus the site number. A region covering several residues with high ratios denotes a conserved predicted compact region during evolution. Currently, the predicted compact regions are regarded as being conserved when the conservation ratios exceed 70% in the same position of the aligned samples [12].

### Contact frequency analysis (F value)

An effective inter-residue potential can be derived from the present inter-residue average distance statistics. In order to analyze the initial folding process of a protein, we performed conformational sampling of a protein in a random state, that is, no secondary or tertiary structure formed, with this potential to identify a residue where initial folding events, such as hydrophobic collapse, occurs [6]. A model of a protein in this study is a Cα bead model. The Metropolis Monte Carlo (MC) method, with the potential energy ε_i,j_ derived from average distance ¯r_i,j_ and its standard deviation σ_i,j,_ was employed in a simulation of protein conformations. The bond and dihedral angles of the initial conformation were randomly selected. During a simulation, the bond and dihedral angles between the residue i and i + 1 are bent and rotated randomly. That is, a simulation is performed starting from a totally random distribution and the restrictions derived from the average distance statistics. The alteration of all the bond and dihedral angles is included in one MC step followed by the Metropolis judgment. We made an assumption that density of the potential between two residues, $\uprho \left({\overline{r}}_{i,j}{\sigma}_{i,j}\right)$, is equal to the probability density derived from the standard Gaussian distribution calculated with its average distance and standard deviation, $\uprho \left({\overline{r}}_{i,j}{\sigma}_{i,j}\right)$, as follows:

3 $$ P\left({\varepsilon}_{i,j}\right)=\rho \left({\overline{r}}_{i,j}{\sigma}_{i,j}\right), $$

where this equation is expressed by Eq. 4:

4 $$ \frac{\mathit{\exp}\left(-\frac{\varepsilon_{i,j}}{kT}\right)}{Z}=\frac{1}{\sqrt{2\pi }{\sigma}_{i,j}}\mathit{\exp}\left\{-\frac{{\left({r}_{i,j}-{\overline{r}}_{i,j}\right)}^2}{2{\sigma}_{i,j}^2}\right\}. $$

We obtain Eqs. 5 and 6 from Eq. 4:

5 $$ -\frac{\varepsilon_{i,j}}{kT}- lnZ=-\ln \left(\sqrt{2\pi }{\sigma}_{i,j}\right)-\frac{{\left({r}_{i,j}-{\overline{r}}_{i,j}\right)}^2}{2{\sigma}_{i,j}^2} $$

6 $$ \frac{\varepsilon_{i,j}}{kT}=\frac{{\left({r}_{i,j}-{\overline{r}}_{i,j}\right)}^2}{2{\sigma}_{i,j}^2}-\ln \frac{Z}{\sqrt{2\pi }{\sigma}_{i,j}}, $$

where kT is set so that the MC acceptance ratio is 0.5. The obtained potential is a harmonic potential to reproduce average distances and standard deviations in the statistics. The significant value in a calculation is the difference between the energy values of conformations, and Z does not appear in the calculation explicitly. Thus, we ignored Z in the calculations.

It is expected that ensembles with reproducible inter-residue average distance statistics using this potential can be obtained. The contact frequency, g(i,j), for each pair of residues is estimated with structures generated from a simulation using the potential energy function. Then the residue contact frequency, g(i,j), in the same range M is normalized, that is, the value corresponding to the z-value in statistical theory. We refer this value as Q(i, j). Here, D(M) is the standard deviation of the contact frequency g(i, j) with two residues separated in range M. These are expressed by Eq. 7 and Eq. 8.

7 $$ D(M)=\sqrt{\frac{\sum_{\left|i-j\right|\in M}{\left(\frac{\sum_{\left|i-j\right|\in M}g\left(i,j\right)}{\sum_{\left|i-j\right|\in M}}-g{\left(i,j\right)}_{\left|i-j\right|\in M}\right)}^2}{\sum_{\left|i-j\right|\in M}}} $$

8 $$ \mathrm{Q}\left(i,j\right)=\frac{g{\left(i,j\right)}_{\left|i-j\right|\in M}-\frac{\sum_{\left|i-j\right|\in M}g\left(i,j\right)}{\sum_{\left|i-j\right|\in M}}}{D(M)} $$

where i and j are the residue numbers.

Finally, we obtain the relative contact frequency, F_i_, by summing the normalized contact frequencies, Q(i,j), from j = 1 to N for each residue i, where N is the total number of residues:

9 $$ {F}_i={\sum}_jQ\left(i,j\right). $$

We call the value F_i_ the “F value”. Residues at the peaks in the plot of F values are expected to be located in the center of many inter-residue contacts, such as a hydrophobic cluster. A region near a peak (within ± 5 residues) [12] of an F-value plot is likely to be significant for folding, especially in the initial stage. We performed ten simulations with 60,000 steps to calculate the average of the F values for residue i. The sampled structures from the very beginning of the simulation were calculated. The location of a peak is regarded as a significant site in an early state of folding. Thus, the definition of a peak is important. According to numerous peaks and valleys are distributed through the F-value profile, the “real” peaks will be defined when the difference in the values of adjacent valleys and a peak is greater than the cut-off value (*F*_*cut*_) as presented in Eq. 10.

10 $$ {F}_{cut}={\left[\frac{1}{N-1}{\sum}_{i=1}^{N-1}{\left({F}_{i+1}-{F}_i\right)}^2\right]}^{\frac{1}{2}}, $$

where F_i_ is the F value of residue i and N is the total residue number, this residue is defined as a peak.

It has been confirmed for several proteins [5–8, 12] that a hydrophobic residue near the F-value peak for a protein tends to form hydrophobic packing in the native structure of a protein. The definition of hydrophobic packing is based on the distance between the heavy atoms of adjacent residues. That is, two conserved hydrophobic residues will be regarded as forming hydrophobic packing when the distance of two residues is less than 5 Å.

### Gō model

A Gō model technique has been applied widely to the folding simulation of a protein [8, 10, 13–15, 34]. A Gō model includes only inter-residue attractive interactions observed in the native structure of a protein and it has been confirmed that a Gō model reproduces the folding process of a protein precisely. In the present study, a coarse-grained Gō model that we developed in our previous articles was used [13, 14] It has been confirmed that our method can reproduce the experimentally observed folding processes of SH3 domain, GB proteins and ferredoxin [13–15]. We briefly explain this method as follows. A Gō model method is.

The potential energy of a structure Γ is defined by Eq. 11.

11 $$ \mathrm{E}\left(\Gamma, {\Gamma}_0\right)=\sum \limits_{angles}{K}_{\theta }{\left({\theta}_i-{\theta}_{i0}\right)}^2+\sum \limits_{dihedral}\left\{{K}_{\upphi}^1\left[-\cos \left({\phi}_i-{\phi}_{i0}\right)\right]+{K}_{\phi}^3\left[-\cos 3{\phi}_i-{\phi}_{i0}\right]\right\}+\sum \limits_{ij}^{NC}{\varepsilon C}_{ij}\left[5{\left(\frac{r_{ij0}}{r_{ij}}\right)}^{12}-{B}_{ij}6{\left(\frac{r_{ij0}}{r_{ij}}\right)}^{10}\right]+\sum \limits_{ij}^{NNC}{\left(\frac{4}{r_{ij}}\right)}^{12} $$

The first, second, third, and fourth terms correspond to the energies of the bond angle, dihedral angle, native interactions, and non-native interactions, respectively. θ, ϕ, r_ij_, NC, and NNC indicate the bond angle, dihedral angle, inter-residue distance, native contacts, and non-native contacts, respectively. The subscript 0 refers to the values related to the native structure. We use the parameters, *K*_*θ*_ = 20*ε*, ${K}_{\upphi}^1=\varepsilon$, and ${K}_{\upphi}^3=0.5\varepsilon$. In this study, an inter-residue native contact is defined when more than one heavy atom pair in two respective residues is within the distance of the sum of the van der Waals radii of two contacting atoms + 1.4 Å [15]. The local contacts, |i − j| < 4, are ignored. The parameter C_ij_ corresponds to the number of inter-heavy atom contacts divided by the average number of inter-heavy atom contacts per residue pair; it indicates the strength of the scaled inter-residue interactions. B_ij_ (Θ_ij_), which is defined by Eqs. 12 and 13, indicates how close a given relative orientation of the i-th and j-th residues is to the native structure.

12 $$ {B}_{ij}\left({\Theta}_{ij}\right)=\left\{\begin{array}{c}\frac{1-{\left({\Theta}_{ij}-{\Theta}_{ij0}\right)}^2}{a_{\Theta}^2}\\ {}0, otherwise\end{array}\right. $$

13 $$ {\Theta}_{ij}={\cos}^{-1}\left(\frac{{\boldsymbol{h}}_i{\boldsymbol{h}}_j}{\mid {\boldsymbol{h}}_i\Big\Vert {\boldsymbol{h}}_j\mid}\right) $$

Θ_*ij*_ indicates the relative orientation of the side chains of the i-th and j-th residues implicitly, namely, the relative angle between *h*_*i*_ and *h*_*j*_. *h*_*i*_ is defined as *r*_*i*, *i* − 1_ + *r*_*i*, *i* + 1_, and *r*_*i*, *i* − 1_ denotes a vector between the i-th and (i - 1) th residues. *h*_*i*_ is used to define a bond vector mimicking the Cβ-Cα vector in the combination of *r*_*i*, *i* − 1_ × *r*_*i*, *i* + 1_ [35]. When Θ_*ij*_ is close to the native value, B_ij_ ~ 1, the residues make a contact. When Θ_*ij*_ exceeds a cutoff value, *B*_*ij*_ ~ 0, the residues cannot make a contact. A cutoff value *a*_Θ_ = 0.6π was used [15]. For the terminal residues, *B*_*ij*_ = 1 because we cannot define the vector *h*_*i*_. We keep θ < π to prevent *h*_*i*_ = 0.

### Simulation

A replica exchange MC simulation was used in the present study [34]. M iterations of pivot moves for randomly selected residues followed by M iterations of crankshaft moves for randomly selected segments are included in one MC step. We set M as the number of residues in a protein. The segment size for a crankshaft move is randomly selected to avoid exceeding half the size of the residue number. After 105 equilibrium steps, we carried out 10^6 simulation steps. We tried to exchange every 100 steps. Various temperature ranges for the proteins were used for the MC simulations: 32 temperatures (kBT/ε) between 0.840 and 1.000 for 1A8D and 32 temperatures between 0.830 and 1.020 for 1TTU. After performing simulations, we calculated the free energy profiles by means of the weighted histogram analysis method (WHAM) from the trajectory at all temperatures [36, 37]. The transition temperature for a protein was determined by the peak of a heat capacity curve.

### Q value and contact frequency map

In this study, an order parameter indicating the closeness of a sampled structure to the native structure of a protein is defined by the following equation.

14 $$ \mathrm{Q}=\frac{native\ contacts\ in\ a\  sampled\ structure}{total\ number\ of\ contacts\ in\ the\ native\ structure\ }. $$

The structural features of sampled structures with a specific Q are expressed using a contact frequency map. A contact frequency map is constructed by plotting the contact frequencies of various pairs of residues on a contact map. Thus, a contact frequency map shows the features of an intermediate state during folding.

## Results

### Secondary-structure-based multiple sequence alignment and conserved hydrophobic residues

**Fig. 4:**
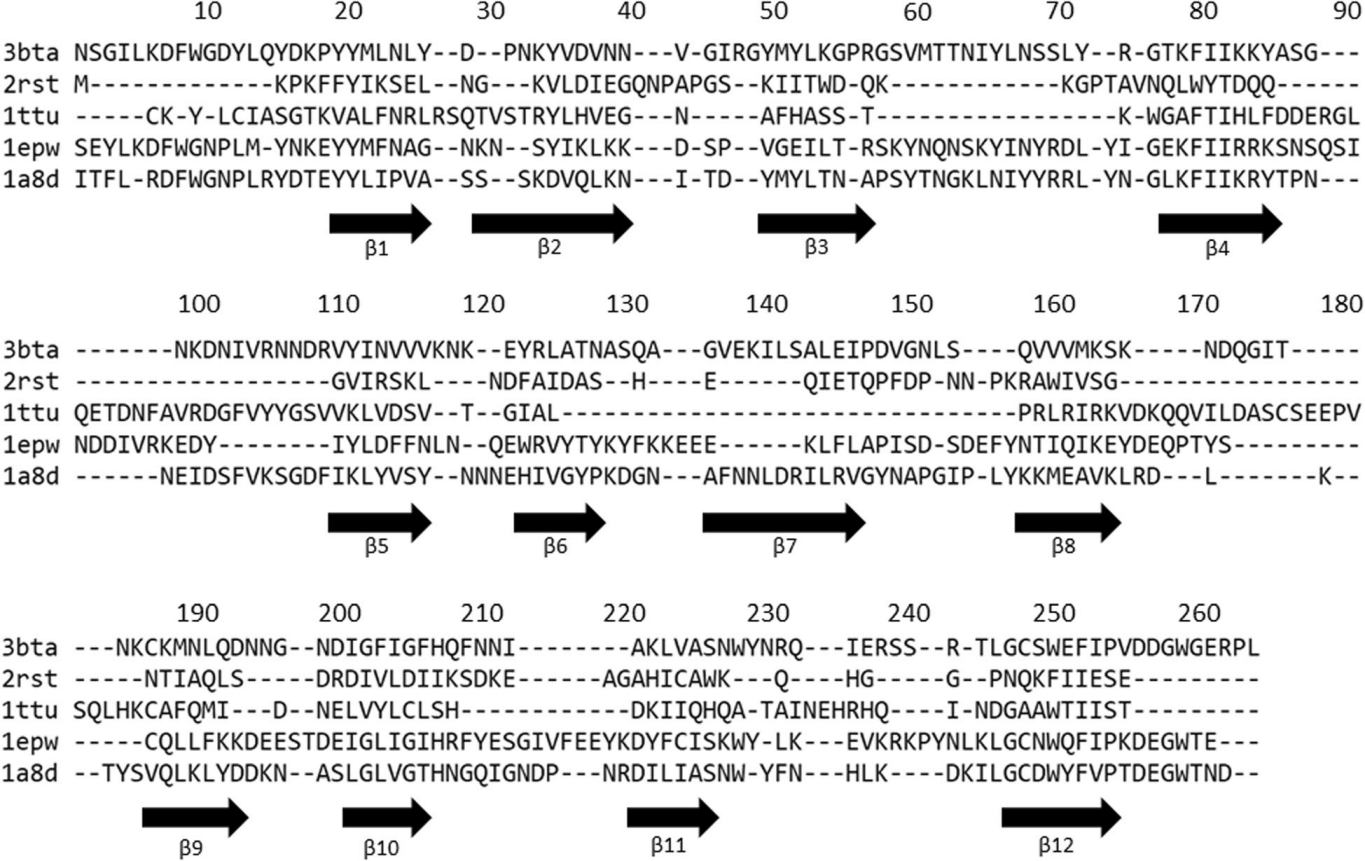
A multiple sequence alignment of irregular and symmetric beta-trefoil proteins. A black arrow indicates a beta-strand

Figure 4 shows the results of using STRAP to align four irregular beta-trefoil proteins with 2RST as a representation of high symmetric beta-trefoil protein. The multiple sequence alignment with ADM results of 26 proteins with high structural symmetry from the previous study [5] and 4 proteins with irregular structures treated in this study based on the secondary structures [31] presented in Fig. S1 in the additional file. We identify conserved hydrophobic residues based on this alignment. In the figure S1, a red bar denotes a region predicted by the ADM for each protein. Brighter red means a higher ƞ value. Fifteen conserved hydrophobic residues identified in the previous study were also observed after four proteins with irregular structures were added. The conserved hydrophobic residues (CHRs) are distributed on all β strands. One or two CHRs are distributed on a β strand. Hereafter, we call a CHR located on the first β-strand as β1 and the two CHRs located on the second β-strand as β2N and β2C and so on. There are two CHRs in β2, β4, and β5, respectively. In 1TTU, β6 and β7 are missing, but an amino acid residue could be confirmed at the corresponding β6 position. We refer to this residue as CHR-β6. However, since β7 does not exist, the number of CHRs was 14 in 1TTU. The blue bar in Fig. S1 showing the conserved compact area of the ADM is presented. The N-terminal and C-terminal ADM predicted regions always correspond to first and third trefoil units. However, it can be seen that the compact areas in the various β-trefoil proteins are diverse.

### ADM analyses and F-value analyses

**Fig. 5:**
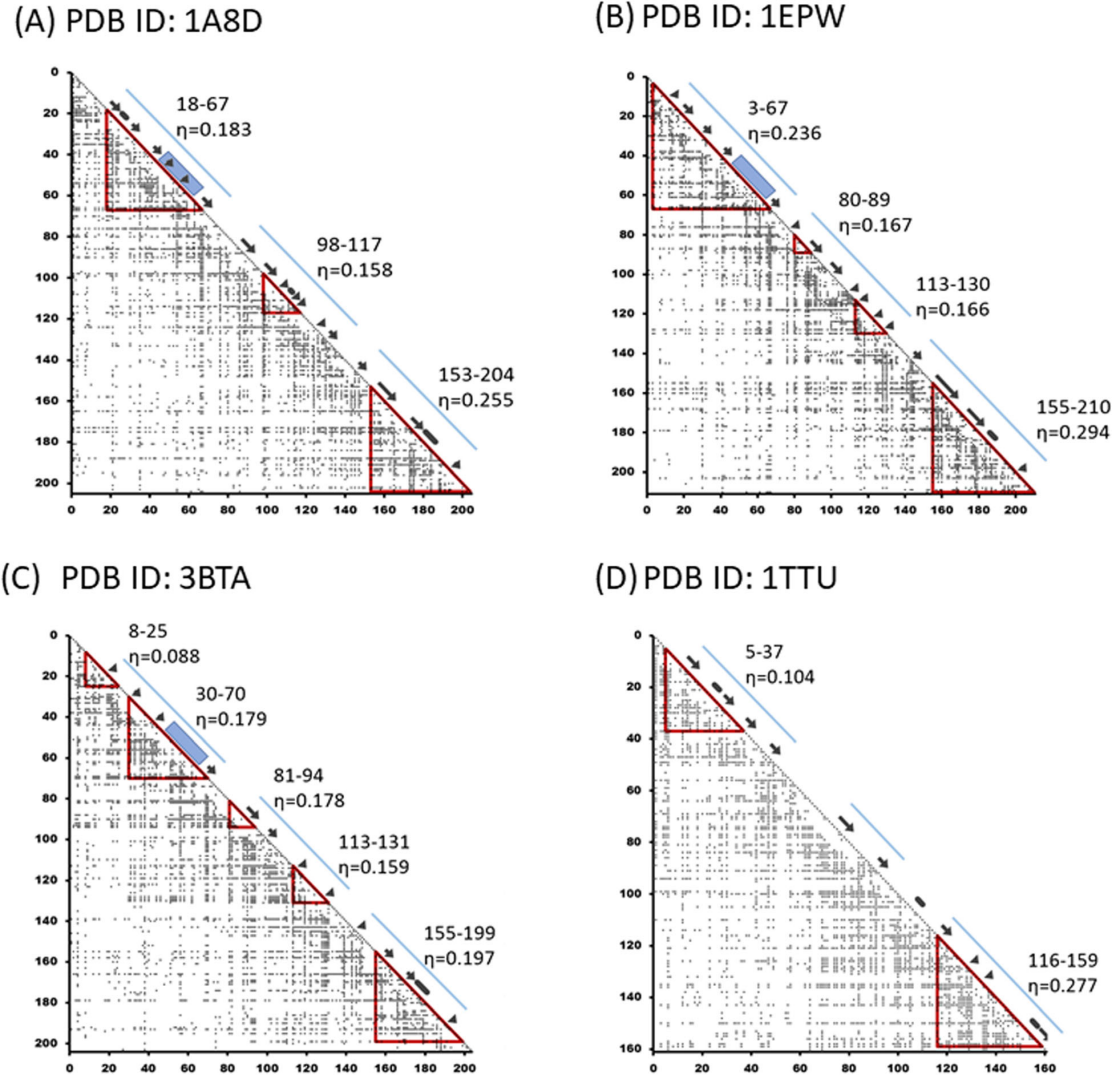
The ADM results of 1A8D (**a**), 1EPW (**b**), 3BTA (**c**), and 1TTU (**d**). A predicted region is enclosed by a red triangle. A black arrow and the blue rectangle along the diagonal indicate a β-strand and the inserted part, respectively. A blue bar along the diagonal indicates a β-trefoil unit

The result of the ADM analysis of 1A8D is shown in Fig. 5a. A compact region having the largest ƞ-value of 0.255 is detected at A153-N204, containing β10-β12. Actually, the compact region I173-N204 exhibits the largest ƞ-value of 0.258. However, that of A153-N204 is 0.255 and almost the same. Thus, we regard A153-N204 as a compact region in this study. There is a compact region with the second largest ƞ-value at residue number Y18-I67, containing β1-β4. This compact area includes the inserted segment. These are the major predicted regions and correspond to trefoil unit 3 and unit 1, respectively. These regions are expected to form a stable compact region in the early stage of folding.

**Fig. 6:**
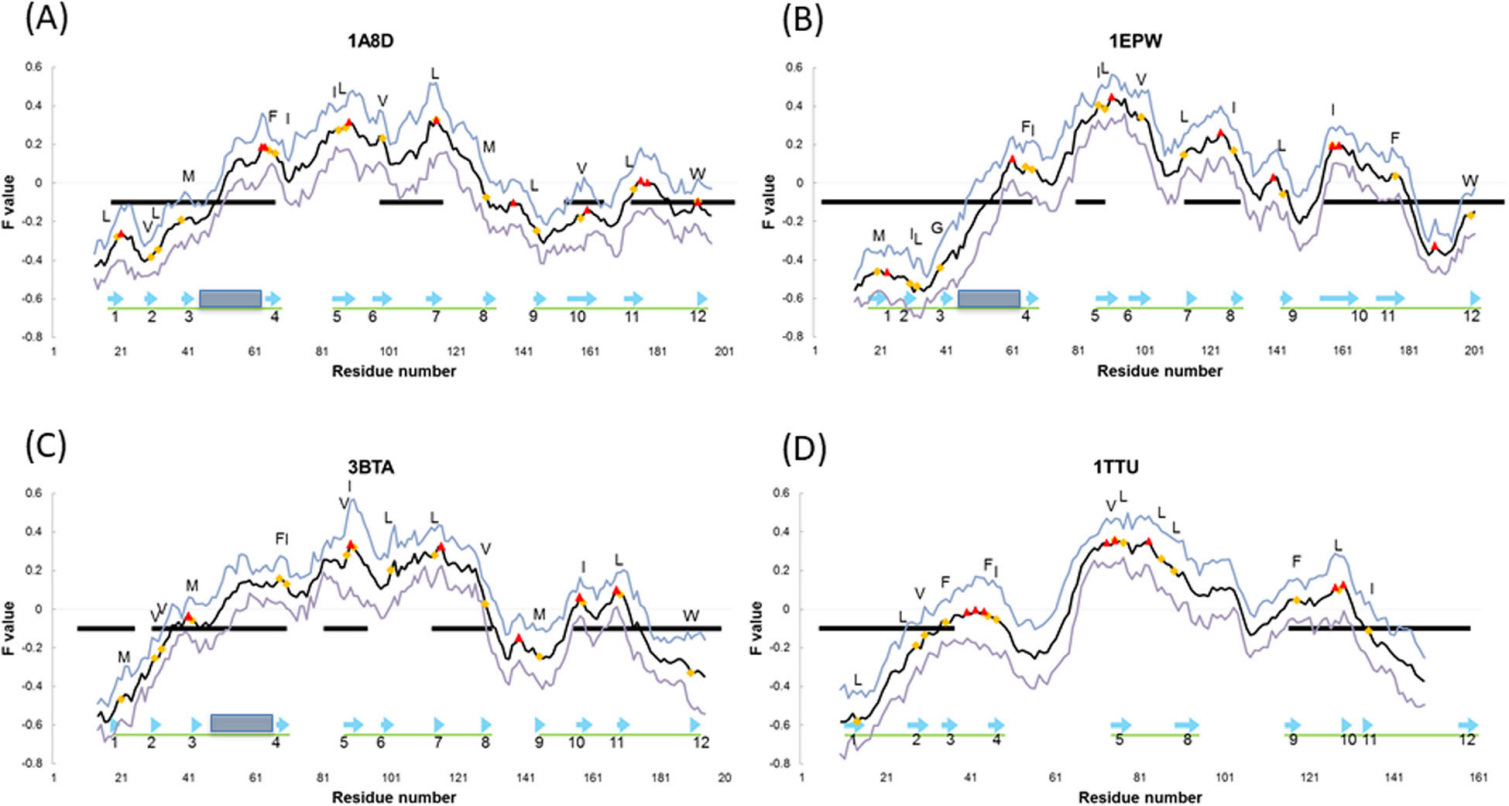
The F-value results of 1A8D (**a**), 1EPW (**b**), 3BTA (**c**), and 1TTU (**d**) (black line). A blue line and a purple line denote + and – standard deviation from the F-value plot. A black bar indicates an ADM-predicted region. A blue arrow and the blue rectangle denote a β-strand and the inserted part. A green bar indicates a β-trefoil unit. A red triangle and an orange diamond indicate a peak of the F-value plot and a conserved hydrophobic residue, respectively

The peaks of the F-value plot appear near β5-β6 and β7 as shown in Fig. 6a. The peak near β7 appears only in 1A8D and 3BTA as mentioned below. The characteristic tendency of the β-trefoil fold in which an F-value plot shows high values at residues in the central trefoil unit was observed in the previous study [5] and is also observed in 1A8D in the current study. The peak of the F-value plot is a residue that is buried inside of a protein and can be regarded as a site that is structured in the early stage of folding [5–8, 12]. It is worth noting that the conserved hydrophobic residues, CHR-β5 and CHR-β7, are close to the F-value peak within ± 5 residues. These results suggest that CHR-β5 and CHR-β7 of the central unit interact with the residues inside and outside the unit and then stabilize I173-N204 and Y18-I67 from the ADM prediction areas.

Figure 5b shows the result of the ADM analysis of 1EPW. A region with residues I155-T210 is predicted as a compact region with the largest ƞ value. This region contains β10-β12. The predicted region with the second largest ƞ-value is Y3-I67, containing β1-β4. This compact area also contains the insertion part in the multiple sequence alignment. These regions also correspond to units 3 and 1 of the β-trefoil, respectively. These regions are expected to form a stable compact region in the early stage of folding. The peaks of the F-value plot appear near β5 and β6 in Fig. 6b. Again, these peaks exhibit the general characteristics [5] observed in β-trefoil proteins. That is, CHR-β5 is close to the F-value peak, as is the case with 1A8D, and similar to 1A8D CHR-β5, it plays a significant role in the early stage of folding.

Figure 5c shows the results of the ADM analysis of 3BTA. For 3BTA, the predicted compact region with the largest ƞ-value is I155-W199, which covers β10-β12 similar to 1EPW. The region with the second highest ƞ-value is Y30-I70, with β2-β4. This compact area also contains the insertion part, as shown in Fig. 2c. Again, these regions correspond to trefoil unit 3 and unit1, respectively. The peaks of the F-value plot present near β5 and β7, as seen in Fig. 6c, which is a common feature of β-trefoil proteins.

As is common with the proteins in the STI-like superfamily, the ADM predicted regions at N-terminus and C-terminus correspond well to the N-terminal and C-terminal trefoil units in each of the three proteins. The results of the present study show a remarkable correspondence of the ADM predicted regions at the N-terminus and C-terminus with those predicted in the previous study [5]. As mentioned, the CHRs of β5 and/or β7 in the central trefoil unit are detected close to the highest peak(s) of the F-value plots for every protein treated in this study. This is also observed in symmetric β-trefoil proteins [5]. These residues are thought to interact with the other residues in the central unit, N-terminal unit, and C-terminal unit in the early stage of folding. Furthermore, an extra peak between β3 and β4 in an F-value plot is observed in Fig. 6a-c. This peak is not observed for the symmetric β-trefoil proteins [5]. Thus, this is also a specific property of proteins in the STI-like superfamily.

Figure 5d presents the result of the ADM analysis of 1TTU. Regions C116-I159, including β9-β12 and C5-A37 containing β1-β3, are predicted to be compact regions with the highest and the second highest ƞ-values. These correspond to unit-3 and unit-1 of the β-trefoil, respectively, which are expected to form stable compact regions in the early stage of folding. No compact area is found in the center, which includes the lacking site, while a peak of the F-value plot appears near β5, as seen in Fig. 6d. We notice that CHR-β5 and CHR-β6 are close to the F-value peak. These residues are considered to play a significant role in folding by interacting with other hydrophobic residues and stabilizing 116–159 and 5–37 of the regions predicted by ADM analysis.

### Analyses of hydrophobic packing among CHRs in a protein based on its 3D structure

**Fig. 7:**
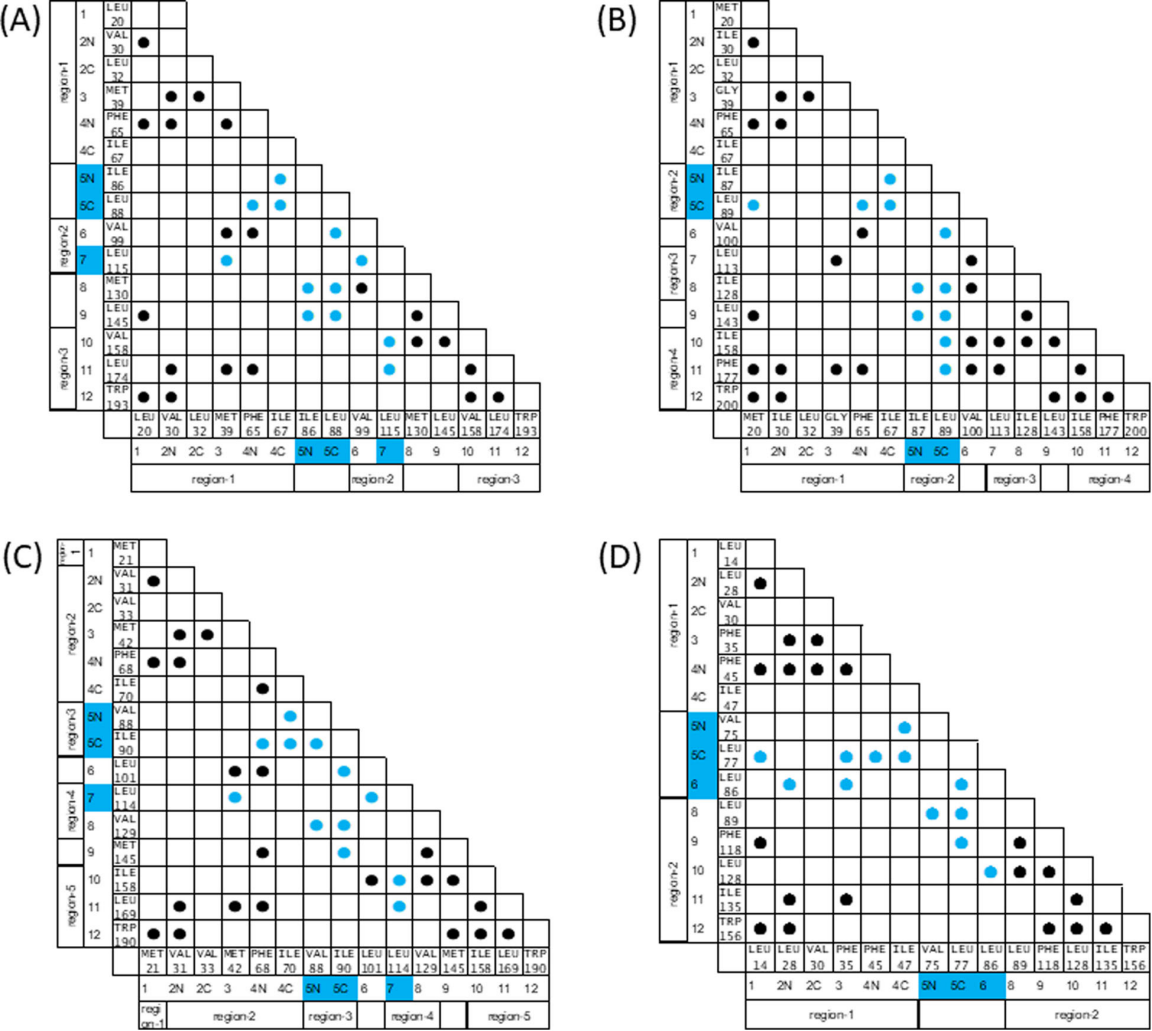
The contact maps of 1A8D (**a**), 1EPW (**b**), 3BTA (**c**), and 1TTU (**d**). A contact formed by conserved hydrophobic residues. Region-1 and so on refer to ADM-predicted regions. A conserved hydrophobic residue highlighted by blue indicates that it is near the highest peak of an F-value plot within ± 5 residues

Next, we analyze the hydrophobic packing among CHRs in a protein based on its 3D structure. In particular, we analyze how CHRs near the peaks of the F-value plot form hydrophobic packing in the native structure of a protein. First of all, three proteins in the STI-like superfamily are treated. Figure 7a shows a contact map of 1A8D taking only conserved hydrophobic residues into account. It is interesting to see that the CHRs in β5 and β7 form several contacts with other CHRs. Considering that CHRs in β5 and β7 are located near the highest F-value peak, these CHRs are important for the structure formation of this protein. CHR-β5C forms packing with CHR-β4N and CHR-β4C in the predicted region-1. Thus, CHR-β5C is particularly significant for the interaction with region-1. On the other hand, CHR-β7 interacts with CHR-β10 in region-3 and with CHR-β11 in region-4 and thus, this residue is thought to be significant for packing with the predicted regions in the C-terminal part.

The F-value plot for 1EPW shows the highest peak at a residue in β5. Figure 7b indicates that CHRs near this peak form some contacts with the CHRs in units 1 and 3. This figure illustrates that CHR-β5C forms packing with CHR-β1, CHR-β4N, and CHR-β4C in the predicted region-1, and CHR-β10 and CHR-β11 in region-4. Thus, CHR-β5C is supposed to be a significant residue for the packing with the predicted regions in the C-terminal and N-terminal parts.

In the 3D structure of 3BTA, CHR-β5C interacts with CHR-β4N and CHR-β4C in the predicted region-2, as shown in Fig. 7c, implying the significance of the interactions formed by CHR-β5C with CHRs in region-2. On the other hand, CHR-β7 interacts with CHR-β3 in region-2 and with CHR-β10 and CHR-β11 in region-5. Therefore, these residues are considered to be significant for packing with the predicted regions in the C-terminal part.

**Fig. 8:**
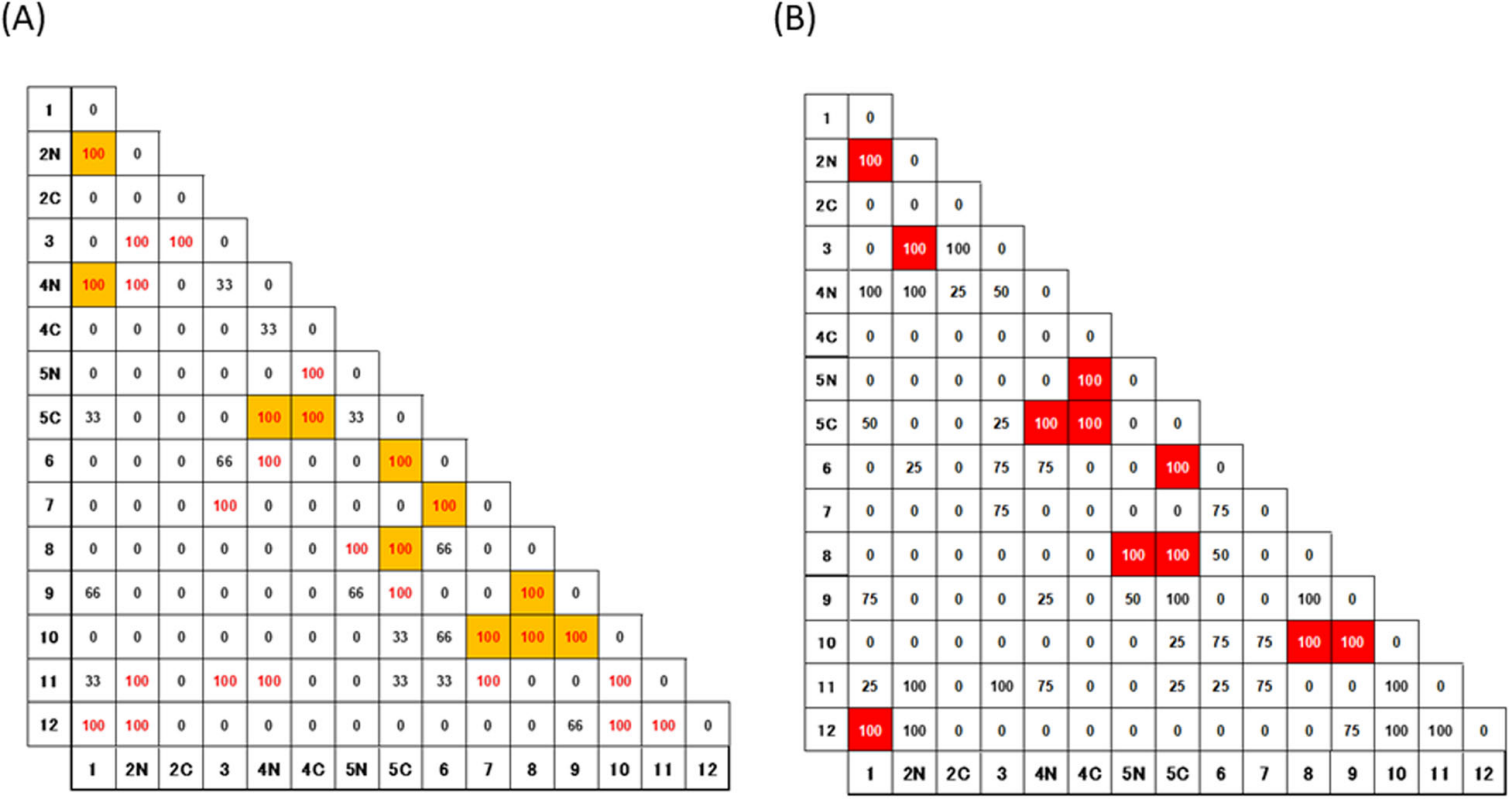
Occurrence ratio of contacts formed by conserved hydrophobic residues. A number shown on the x-axes and y-axes denotes a conserved hydrophobic residue. The occurrence ratio is indicated by a percentage in each cell. **a** Conserved hydrophobic residues of three irregular beta-trefoil proteins in STI-like superfamily, including 1A8D, 1EPW and 3BTA. Red color indicates 100%. An orange cell is a contact that is preserved even in a β-trefoil with high structural symmetry. **b** Conserved hydrophobic residues of all four irregular beta-trefoil proteins used in this study. A cell highlighted by red denotes a 100% contact observed in these proteins and also observed in 26 proteins treated in the previous study [5].

There are 26 common contacts formed by CHRs in three proteins of the STI-like superfamily. Figure 8a shows the occurrence of contact formations between the CHRs in three proteins. The number in each cell of Fig. 8a denotes the percentage of each contact in the three proteins. Among them, 11 contacts formed by CHRs are also observed in 26 β-trefoil proteins with perfect three-fold symmetry [5]. In particular, the contacts formed by CHR-β5C with CHR-β4N, CHR-β4C, CHR-β5N, CHR-β6, CHR-β8, and CHR-β9 are always observed in the three proteins. That is, CHR-β5C seems to be a hub of the interaction network in the three proteins.

In the case of 1TTU, as shown in Fig. 7d, CHR-β5C interacts with CHR-β1 in predicted region-1, and with CHR-β8 and CHR-β9 in predicted region-2. That is, CHR-β5C plays a role in connecting the N-terminal and C-terminal regions and is considered to be significant in the formation of the whole 3D structure. On the other hand, CHR-β7 forms packing with CHR-β2N and CHR-β3 in predicted region-1 and with CHR-β8 and CHR-β10 in predicted region-2. CHR-β7 can be also a significant residue in the 3D-structure formation.

Let us compare the hydrophobic packing in the symmetric β-trefoil proteins with that in the proteins with irregular structures (Fig. 8b). The commonly appearing contacts in both protein groups are highlighted by red. As a result, 11 common contacts are confirmed (contacts appearing more than 95% were counted). Four of 11 contacts are formed by CHR-β5C. CHR-β5C almost always forms packing with CHR-β4N and CHR-β4C in trefoil unit-1 and with CHR-β6 and CHR-β8 in trefoil unit-2. Thus, CHR5C is considered to play a role as the core of the central unit formation connecting unit-1 and the central unit. CHR-β8 forms packing with CHR-β5C in trefoil unit-2 and with CHR-β9 and CHR-β10 in trefoil unit-3, and is therefore the residue connecting trefoil unit-3 and the central trefoil unit. CHR-β1 forms packing with CHR-β2N and CHR-β4 N in trefoil unit-1. For this reason, CHR-β1 seems to be the significant residue in forming trefoil unit-1. CHR-β5N shows two common contacts with four irregular structure proteins. That is, CHRβ5 is commonly significant for symmetric as well as irregular β-trefoil proteins.

It is interesting to see the interaction between CHR-β6 and CHR-β7 conserved in 26 β-trefoil proteins with the high symmetry structures and three STI-like superfamily proteins. However, this packing disappears in 1TTU because β7 is missing. Instead of this packing, a new interaction between CHR-β6 and CHR-β10 is observed. It is thought that this interaction between CHR-β6 and CHR-β10 in 1TTU compensates for the missing interaction between CHR-β6 and CHR-β8.

### Gō-model simulation

**Fig. 9:**
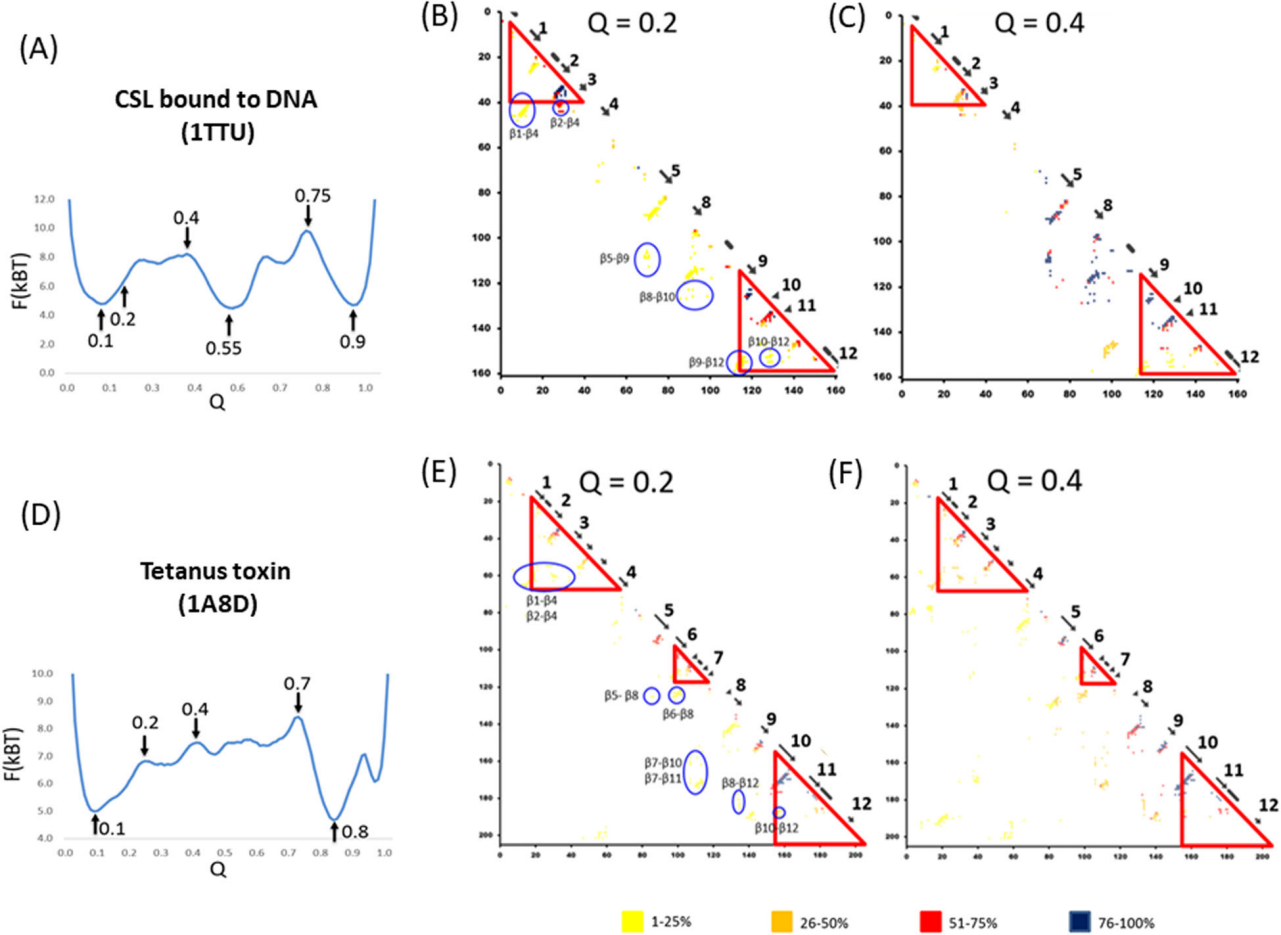
Gō-model simulation results of 1TTU and 1A8D. The upper figures are the results of 1TTU, including (**a**) Free energy profile at T = 0.938, (**b**) Contact frequency map at Q = 0.20 and (**c**) Contact frequency map at Q = 0.30. The lower figures are the results of 1A8D, including (**d**) Free energy profile at T = 0.921, (**e**) Contact frequency map at Q = 0.20 and (**f**) Contact frequency map at Q = 0.40. The contacts enclosed by a blue circle are the long-range contacts observed in an initial state of folding

In this section, we present the results of the Gō-model simulations. Figure 9a presents the free-energy profile at about T = 0.94 of 1TTU. We identify three clear wells at Q = 0.10, 0.55, and 0.9, corresponding to denatured stable, intermediate, and native states. In the free energy profile of 1TTU (Fig. 9a), two major transition states are identified at Q = 0.40 and 0.75. Figure 9b is the contact frequency map at Q = 0.20. In this figure, the contacts between β2 and β3, β2 and β4 and between β9 and β10 are frequently formed at Q = 0.20. These highly frequent contacts correspond to the ADM predicted regions 5–37 and 116–159, which corresponds to just before the first transition state. That is, an ADM-predicted compact region can be regarded as a compact region in the early stage of folding. The time course of folding and the Q value do not coincide exactly. However, a small value of Q is considered to correspond to the early stage of folding. The contact map for Q = 0.4 in Fig. 9c reveals that the long range contacts (k ≥ 16, k = |i-j|, where i and j refer to the residue numbers) [14] start to form among CHRs in β3, β4, β5, β6, and β10 near the F-value plot peaks, namely, V35, F45, I47, V75, L77, L86, and L128, respectively.

Next, we present the result of Gō-model simulation for 1A8D. In Fig. 9d, the free energy profile is presented. In Fig. 9e, in order to focus the initial folding process, Q = 0.2 corresponds to the state just before the first transition state in the folding process. The N-terminal and C-terminal regions containing highly frequent contacts correspond to the ADM-predicted regions 18–67 and 153–204 at Q = 0.2. In other words, an ADM-predicted compact region can be regarded as a compact region in the early stage of folding. The contact map for Q = 0.4 reveals that the long-range contacts between the ADM-predicted regions are formed by CHRs in β1, β4, β5, β7, β10, β11, and β12 near the F-value peaks, that is, L20, F65, I67, I86, L88, L115, V158, L174, W193, respectively.

The second transition state presents at Q = 0.4. From the observation of the contacts at Q = 0.4, the CHRs in trefoil unit-2 tend to form contacts with the CHRs in trefoil unit-1 and unit-3 rather than within trefoil unit-2.

## Discussion

In the present study, the folding initiation sites in the β-trefoil proteins with irregular structures were predicted from their amino acid sequences and further analyses were made regarding the packing formed by CHRs based on the 3D structures. These results were verified by Gō-model simulations. As a result, CHRs in the central trefoil unit with high F-value peaks interact with CHRs within the trefoil unit and with CHRs in other trefoil units, followed by the stabilization and structural formation of ADM-predicted regions corresponding to the N-terminal and C-terminal trefoil units. For example, in the folding of 1A8D, the hydrophobic packing formed by CHRs in the ADM-predicted region-1 (β1-β4) and region-3 (β10-β12) play significant roles in forming the N-terminal and C-terminal units, respectively, as indicated in Fig. 5a and b. CHRs in β5-β7 interact with those in region-1 and region-3 to form the whole 3D structure. Similarly, in 1TTU, the C-terminal unit is formed by the interactions among CHRs in β9-β12, while the N-terminal unit is formed by CHRs in β1-β3. Furthermore, CHRs in β5-β8 form hydrophobic packing, and the whole 3D structure is constructed. In the results of the Gō-model simulation for 1TTU, the contacts between β2 and β3, β9 and β10, and β10 and β11 are observed at the initial stage of folding, that is, Q = 0.2. The locations of these contacts correspond well to the ADM-predicted regions. After the formation of these contacts, when Q = 0.3, local and nonlocal contacts are observed in the region around β5 and β8. This region corresponds to the broad part with high values in the F-value plot. In the Gō-model results for 1A8D, the native contacts between β2 and β3, β6 and β7, and β10 and β11 form with high occurrence at Q = 0.2, with these regions corresponding well to the ADM-predicted regions. Furthermore, nonlocal contacts are observed between β5 and β7. The CHRs forming these contacts correspond to those around the peaks in the F-value plots. The native contacts appear at the states with an approximate Q of 0.2, that is, from the stable denatured state to the first transition state in the Gō-model simulations in the ADM-predicted region. Moreover, nonlocal contacts appear at the first transition state with Q = 0.3, corresponding to CHRs around the peaks of the F-value plot. This result also implies that the predictions from the sequences coincide with the Gō-model analyses.

These facts suggest that the information about the initial stage of folding of a protein can be extracted from its sequence by ADMs and F-value analyses. A Gō-model simulation in the present study demonstrates that CHRs that form contacts are mainly within an ADM-predicted region in the early stage of folding. These CHRs are located around F-value peaks. Contacts by CHRs between ADM-predicted regions start to form with increasing Q value, as is the case for 1A8D in Fig. 9c. These results confirmed that CHRs play a significant role in protein folding.

As an attempt, we perform the predictions of disordered regions based on our new technique [38]. The results are presented in Figure S3 in the additional file. In general, these four β-trefoil proteins are regarded as ordered proteins. For 1TTU, the short regions, 36–41, 53–58 and 149–150 are predicted as disordered regions. The region 36–41 corresponds to β3, 53–58 corresponds to the irregular part, and 149–150 is a part which do not form major contacts. (It should be notified that similar regions are predicted as disorder in 1A8D, 1EPW and 3BTA because these are classified in the same superfamily.) Comparing the Gō model result in Fig. 9, 36–41 forms contacts with β2 at Q = 0.2, that is, relatively early stage of folding. This part is considered to form an ordered structure by the interaction with β2. The regions 53–58 and 149–150 may rather fluctuate in the native structure. For 1A8D, again the short regions, 10–14, 46–48, 105–108, and 200–205 are predicted as disorder. 10–14 in the N-terminus is the part not making major contacts. 46–48 corresponds to the irregularly inserted part. 200–205 is the C-terminus. Thus, these regions seem not to form rigid 3D structures. The interactions regarding these parts do not appear in the Gō model result in Fig. 9.

In this study, the common 14 contacts formed by CHRs in the β-trefoil proteins with high symmetry could be defined. According to this result, 11 of the 14 common contacts are also observed in the β-trefoil proteins with irregular structures. Thus, these 11 contacts seem to be especially significant in the formation of the β-trefoil scaffold. Two of four deficient contacts, namely, the contacts between β6 and β7 and between β7 and β10, are caused by the deficiency of β7 in 1TTU. The corresponding contacts are observed in the other three proteins, that is, 1A8D, 1EPW, and 3BTA. These corresponding contacts seem to be significant but not indispensable for β-trefoil structure formation. The other contact between β6 and β8 is not observed in 3BTA and 1TTU. It should be noted that the residues corresponding to this contact pair are close to each other in 3BTA. In 1TTU, these corresponding two residues are located within three residues along the sequence. Therefore, we do not take these two residues into account.

In the three STI-like superfamily proteins, the ADM-predicted regions always contain the insertion parts, suggesting that this insertion does not disturb the N-terminal trefoil-unit formation. In the Gō-model simulations, some contacts formed by the residues in the insertion part are observed in the early stage of folding (Q = 0.2).

The native contacts in 1TTU show that β6 interacts with β10 in the C-terminal side (Fig. 9). This contact is not frequently observed in the β-trefoil proteins with high symmetry. That is, β6 assumes the role of the deficient β7.

In the present study, the results from Gō-model simulations coincide well with the predictions made by the ADMs and F-value analyses based on the amino acid sequence information. ADMs and F-value analyses can decode the folding information from the amino acid sequences of not only the β-trefoil proteins with high symmetry but also of those with irregular structures.

Finally, we mention that we are currently thinking to make a webserver to perform present analyses for various proteins in near future.

## Conclusion

The present study demonstrates that the ADM-predicted regions in the N-terminal and C-terminal parts correspond well to the N-terminal and C-terminal β-trefoil units of a β-trefoil protein with an irregular structure. The insertion part is always in the N-terminal ADM-predicted region for each of the three proteins in the STI-like superfamily. In contrast, a loop region tends to be excluded from an ADM-predicted region for a highly symmetrical β-trefoil protein [5]. These facts imply that such an insertion in a STI-like superfamily protein plays a role in its folding.

The property of the peak(s) of the F-value plot for a highly symmetrical β-trefoil protein appearing in the central region is also observed for a β-trefoil protein with an irregular structure. Thus, this property is quite common in the space of β-trefoil protein 3D structures.

The analyses of the packing formed by CHRs in the native structures reveal 11 common interactions, which are considered to be significant factors in constructing the β-trefoil scaffold. In particular, we revealed the central role of CHR-β5N and CHR-β5C in forming both symmetric and irregular β-trefoil scaffolds.

Thus, our results suggest that the folding properties of highly symmetrical and irregular β-trefoil proteins are basically conserved, and our sequence-based techniques can decode these properties from their amino acid sequences.

## Supplementary information

**Additional file 1.**

## Data Availability

Data sharing is not applicable to this article as no datasets were generated or analysed during the current study.

## Abbreviations

ADM: Average Distance Map
RDM: Real Distance Map
MC: Metropolis Monte Carlo
STRAP: STRuctural Alignments of Proteins
CHR: Conserved Hydrophobic Residue

## References

[1] McLachlan AD (1979). Three-fold structural pattern in the soybean trypsin inhibitor (Kunitz). J Mol Biol.

[2] Orengo CA, Jones DT, Thornton JM (1994). Protein superfamilies and domain superfolds. Nature.

[3] Ponting CP, Russell RB (2000). Identification of distant homologues of fibroblast growth factors suggests a common ancestor for all beta-trefoil proteins. J Mol Biol.

[4] Sweet RM, Wright HT, Janin J, Chothia CH, Blow DM (1974). Crystal structure of the complex of porcine trypsin with soybean trypsin inhibitor (Kunitz) at 2.6-a resolution. Biochemistry.

[5] Kirioka T, Aumpuchin P, Kikuchi T (2017). Detection of folding sites of β-trefoil fold proteins based on amino acid sequence analyses and structure-based sequence alignment. J Proteomics Bioinform.

[6] Ichimaru T, Kikuchi T (2003). Analysis of the differences in the folding kinetics of structurally homologous proteins based on predictions of the gross features of residue contacts. Proteins.

[7] Matsuoka M, Fujita A, Kawai Y, Kikuchi T (2014). Similar structures to the E-to-H helix unit in the globin-like fold are found in other helical folds. Biomolecules.

[8] Matsuoka M, Sugita M, Kikuchi T (2014). Implication of the cause of differences in 3D structures of proteins with high sequence identity based on analyses of amino acid sequences and 3D structures. BMC Research Notes.

[9] Ishizuka Y, Kikuchi T (2011). Analysis of the local sequences of folding sites in β sandwich proteins with inter-residue average distance statistics. The Open Bioinformatics Journal.

[10] Aumpuchin P, Kikuchi T (2019). Prediction of folding mechanisms for Ig-like beta sandwich proteins based on inter-residue average distance statistics methods. Proteins: Structure, Function, and Bioinformatics.

[11] Matsuoka M, Kikuchi T (2014). Sequence analysis on the information of folding initiation segments in ferredoxin-like fold proteins. BMC Struct Biol.

[12] Nakashima T, Kabata M, Kikuchi T (2017). Properties of amino acid sequences of lysozyme-like superfamily proteins relating to their folding mechanisms. J Proteomics Bioinform.

[13] Sugita M, Kikuchi T (2013). Incorporating into a Calpha go model the effects of geometrical restriction on Calpha atoms caused by side chain orientations. Proteins.

[14] Sugita M, Kikuchi T (2013). Analyses of the folding properties of ferredoxin-like fold proteins by means of a coarse-grained go model: relationship between the free energy profiles and folding cores. Proteins.

[15] Sugita M, Matsuoka M, Kikuchi T (2015). Topological and sequence information predict that foldons organize a partially overlapped and hierarchical structure. Proteins.

[16] Koga N, Takada S (2001). Roles of native topology and chain-length scaling in protein folding: a simulation study with a Gō-like model. J Mol Biol.

[17] Ferguson A, Liu Z, Chan HS (2009). Desolvation barrier effects are a likely contributor to the remarkable diversity in the folding rates of small proteins. J Mol Biol.

[18] Chavez LL, Onuchic JN, Clementi C (2004). Quantifying the roughness on the free energy landscape: entropic bottlenecks and protein folding rates. J Am Chem Soc.

[19] Clementi C, Nymeyer H, Onuchic JN (2000). Topological and energetic factors: what determines the structural details of the transition state ensemble and “en-route” intermediates for protein folding? An investigation for small globular proteins. J Mol Biol.

[20] Larriva M, Prieto L, Bruscolini P, Rey A (2010). A simple simulation model can reproduce the thermodynamic folding intermediate of apoflavodoxin. Proteins: Structure, Function and Bioinformatics.

[21] Karanicolas J, Brooks CL (2003). Improved Gō-like models demonstrate the robustness of protein folding mechanisms towards non-native interactions. J Mol Biol.

[22] Karanicolas J, Brooks CL (2002). The origins of asymmetry in the folding transition states of protein L and protein G. Protein Sci.

[23] Clementi C, Garcıa AE, Onuchic JN (2003). Interplay among tertiary contacts, secondary structure formation and side-chain packing in the protein folding mechanism: all-atom representation study of protein L. J Mol Biol.

[24] Berman HM, Westbrook J, Feng Z, Gilliland G, Bhat TN, Weissig H, Shindyalov IN, Bourne PE (2000). The protein data Bank. Nucleic Acids Res.

[25] Swaminathan S, Eswaramoorthy S (2000). Structural analysis of the catalytic and binding sites of Clostridium botulinum neurotoxin B. Nat Struct Biol.

[26] Lacy DB, Tepp W, Cohen AC, DasGupta BR, Stevens RC (1998). Crystal structure of botulinum neurotoxin type a and implications for toxicity. Nat Struct Biol.

[27] Kovall RA, Hendrickson WA (2004). Crystal structure of the nuclear effector of notch signaling, CSL, bound to DNA. EMBO J.

[28] Knapp M, Segelke B, Rupp B (1998). The 1.61 Angstrom structure of the tetanus toxin. Ganglioside binding region: solved by MAD and MIR phase combination. Am Cryst Assoc.

[29] Kikuchi T, Nemethy G, Scheraga HA (1988). Prediction of the location of structural domains in globular proteins. J Protein Chem.

[30] Shindyalov IN, Bourne PE (1998). Protein structure alignment by incremental combinatorial extension (CE) of the optimal path. Protein Eng.

[31] Gille C, Frömmel C (2001). STRAP: editor for STRuctural alignments of proteins. Bioinformatics.

[32] Tsigelny IF (2002). Protein structure prediction: Bioinformatic approach, vol. 3.

[33] Dobson CM (2003). Protein folding and misfolding. Nature.

[34] Mitsutake A, Sugita Y, Okamoto Y (2001). Generalized-ensemble algorithms for molecular simulations of biopolymers. Biopolymers.

[35] Sulkowska JI, Cieplak M (2008). Selection of optimal variants of go-like models of proteins through studies of stretching. Biophys J.

[36] Ferrenberg AM, Swendsen RH (1988). New Monte Carlo technique for studying phase transitions. Phys Rev Lett.

[37] Ferrenberg AM, Swendsen RH (1989). Optimized Monte Carlo data analysis. Phys Rev Lett.

[38] Shimomura T, Nishijima K, Kikuchi T (2019). A new technique for predicting intrinsically disordered regions based on average distance map constructed with inter-residue average distance statistics. BMC Struct Biol.

