# Supplementary material for: Analyses of the folding sites of irregular β-trefoil fold proteins through sequence-based techniques and Gō-model simulations
Source: BMC Mol Cell Biol. 2020 Jul 21;21:28. doi: 10.1186/s12860-020-00271-4 (PMC7477875; doi:10.1186/s12860-020-00271-4)
Supplement: Supplementary file 1 — Additional file 1. [file 12860_2020_271_MOESM1_ESM.docx]

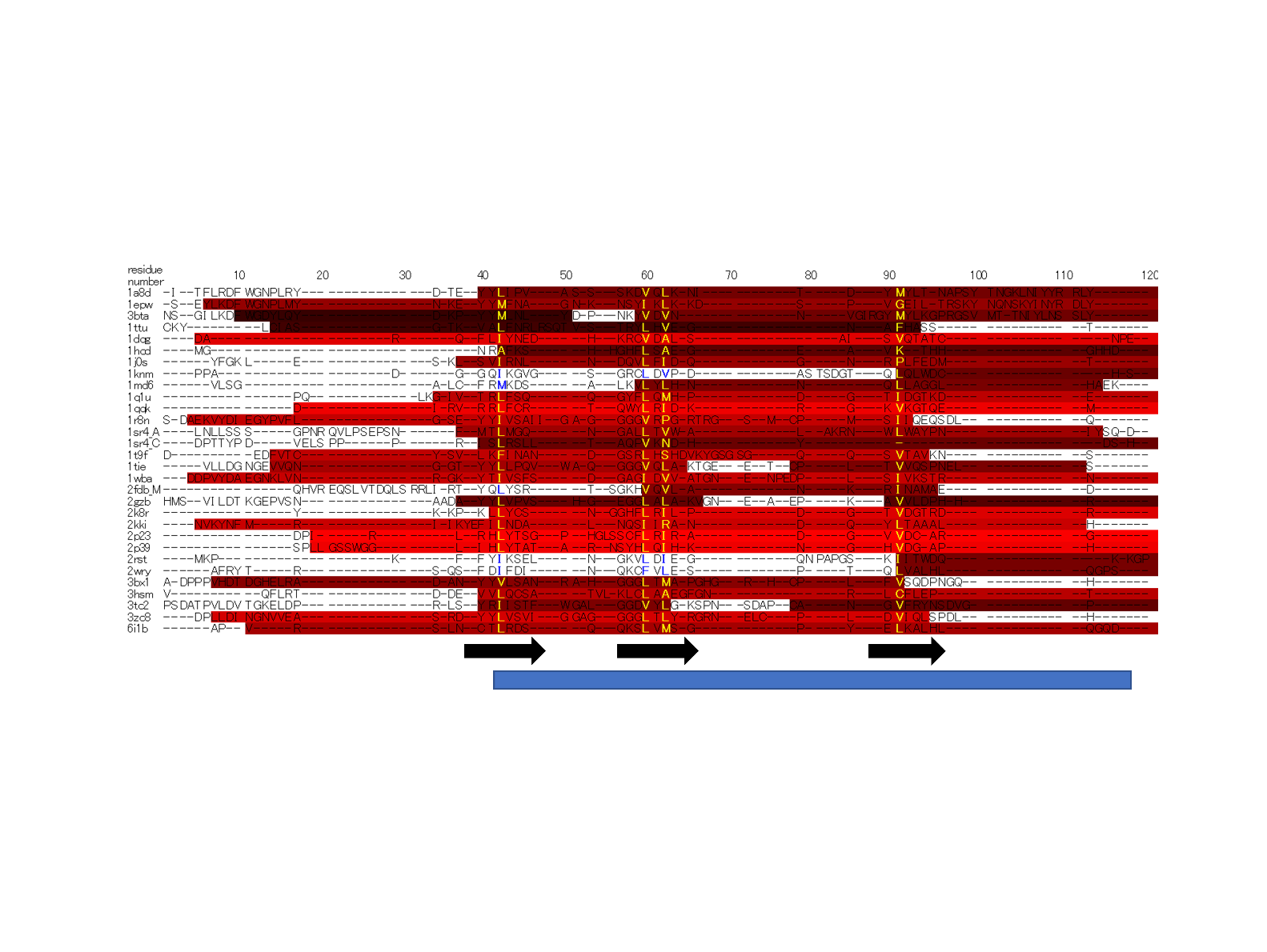


**Fig. S1 Structure-based multiple sequence alignment of selected proteins.** The predicted compact region by ADM is indicated by a red bar. The brighter red denotes a higher compact density. The conserved hydrophobic residues in the predicted compact region are yellow, and a blue letter is a residue out of the predicted compact region. A black arrow represents beta-strand. A blue bar indicates the conserved predicted compact region when the conservation over than 70% of the aligned site.


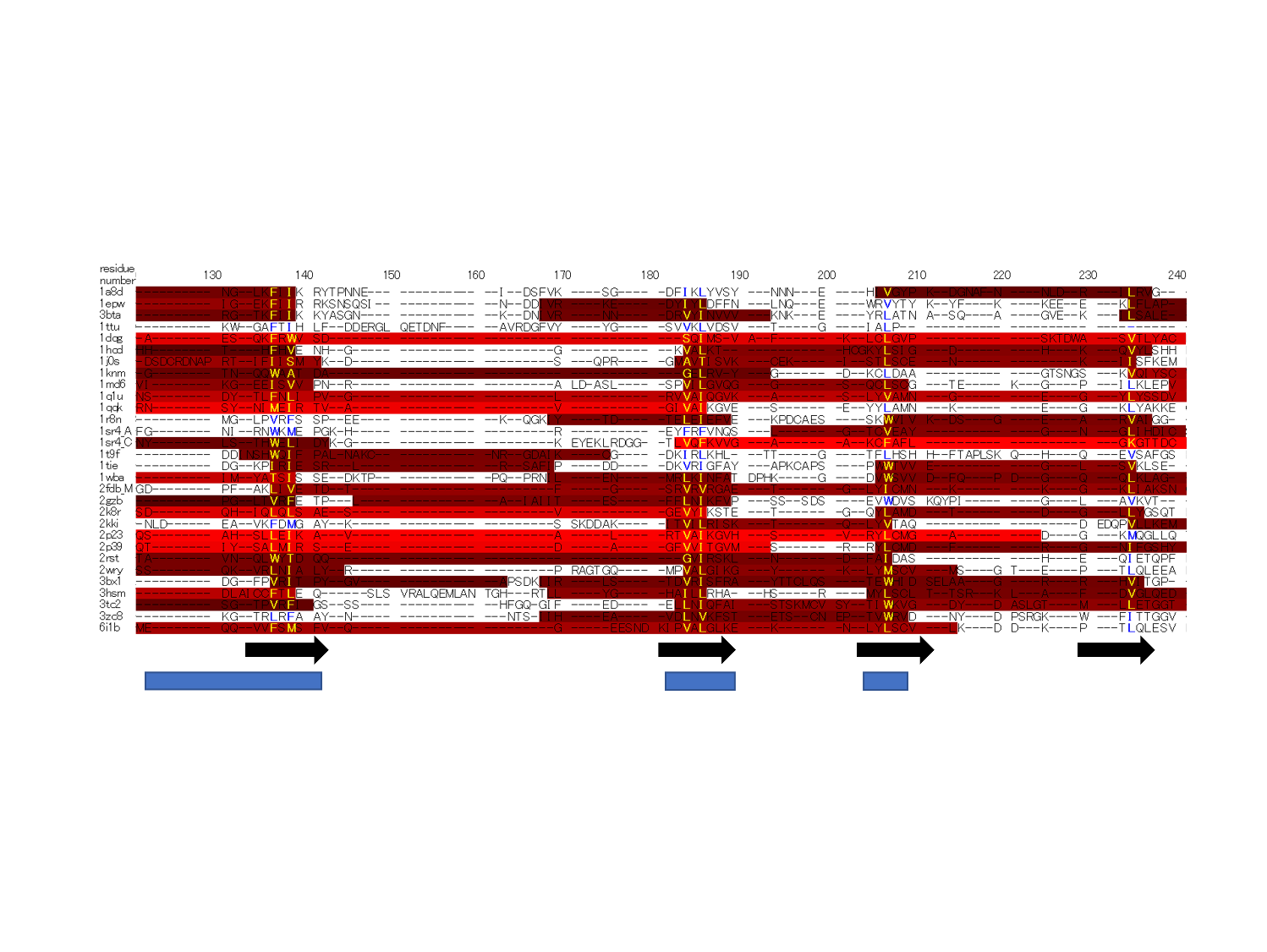


**Fig. S1 Continued**


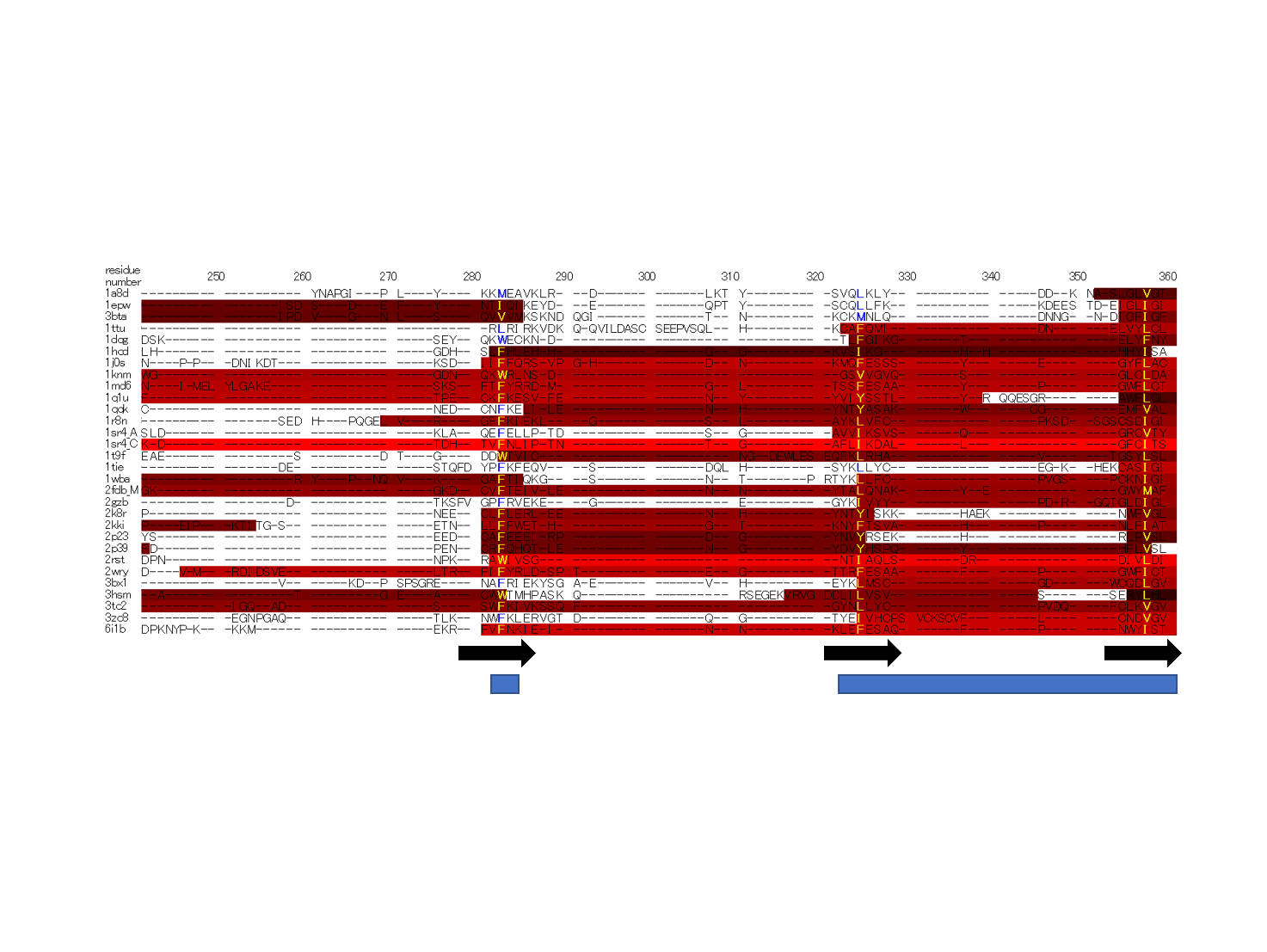


**Fig. S1 Continued**


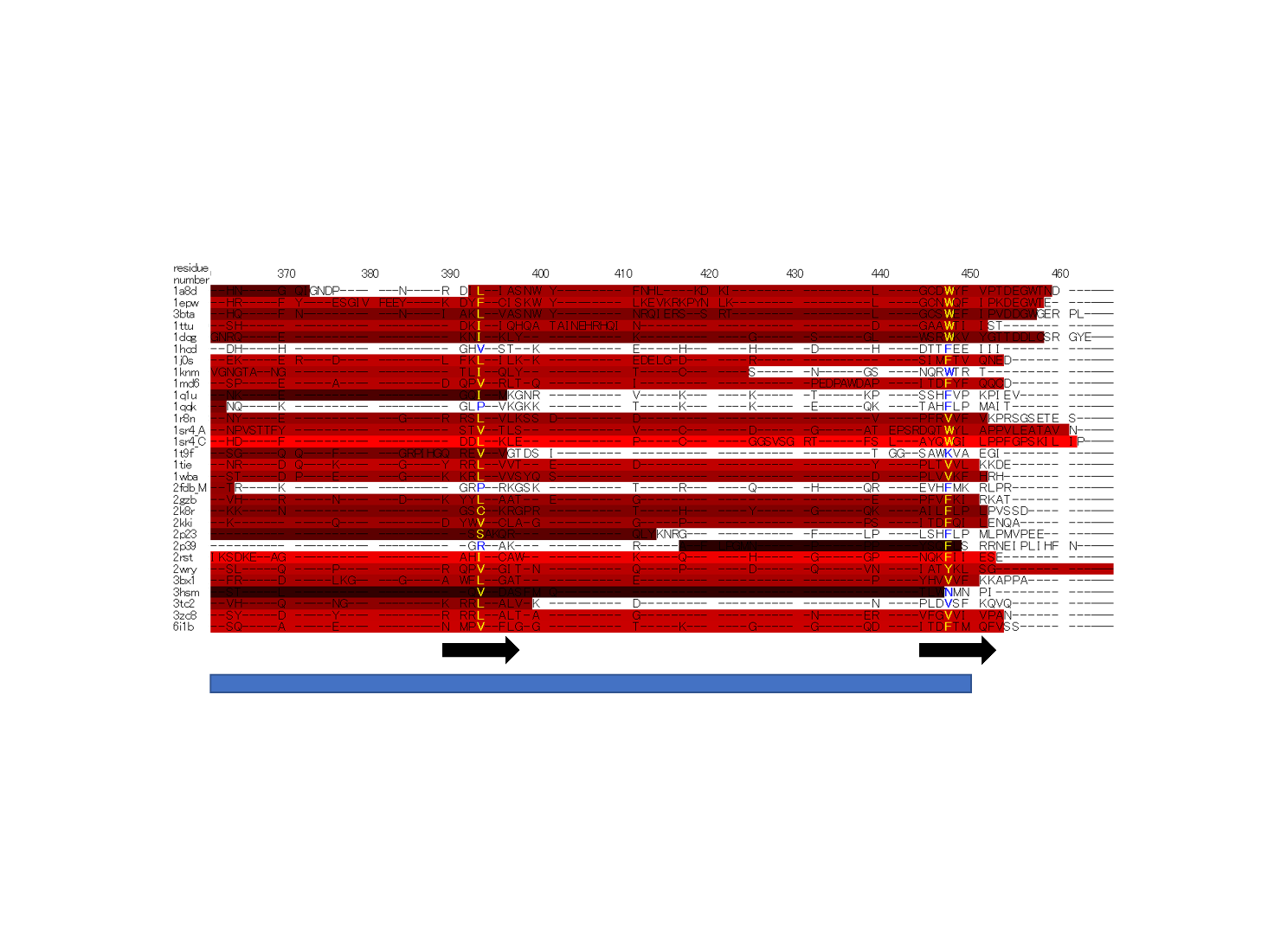


**Fig. S1 Continued**


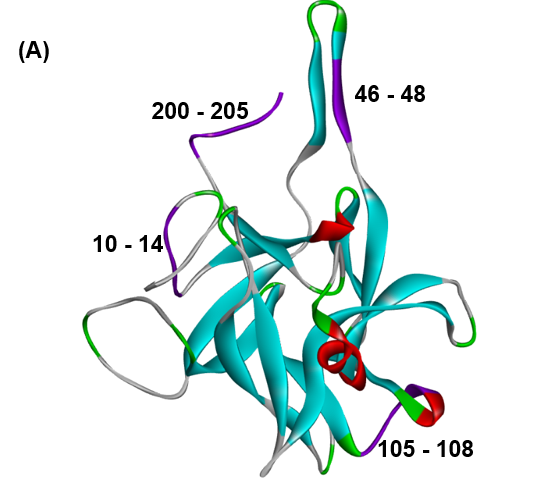

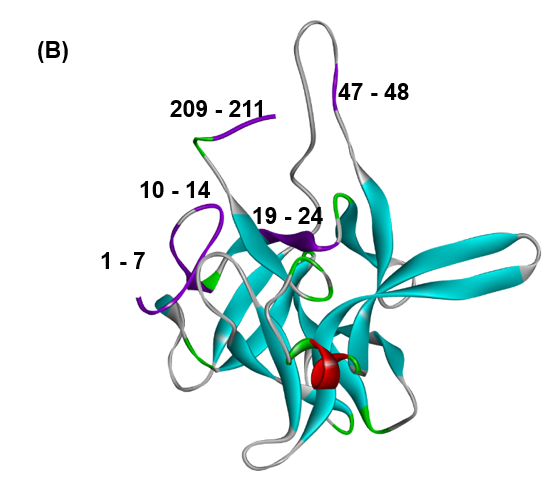


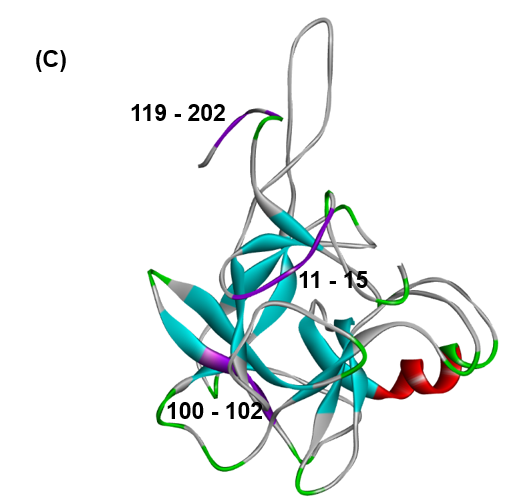

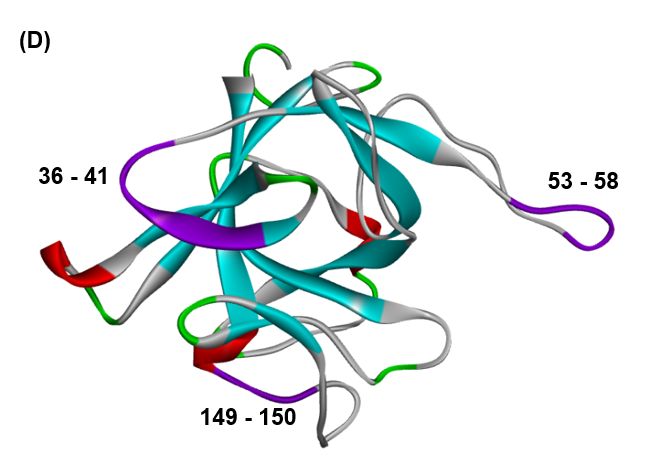


**Fig. S2 Predicted disordered regions in 1A8D(A), 1EPW(B), 3BTA(C) and 1TTUY(D) based on the method proposed by Shimomura et al.** A predicted disordered region is colored by purple. Similar regions are predicted as disorder in 1A8D, 1EPW and 3BTA because these are classified in the same superfamily.
